# An Orc1 initiator-specific motif (ISM)-related region limits ORC–ssDNA binding and promotes replication origin specificity in budding yeast

**DOI:** 10.3389/fmicb.2026.1778270

**Published:** 2026-04-22

**Authors:** Hironori Kawakami, Takeaki Chichibu, Ryuya Muraoka, Shota Kanamoto, Kanako Asada, Eiji Ohashi, Takuya Kurihara, Tsutomu Katayama

**Affiliations:** 1 Laboratory for Systems Immunology, Faculty of Pharmaceutical Sciences, Sanyo-Onoda City University, Yamaguchi, Japan; 2 Department of Molecular Biology, Graduate School of Pharmaceutical Sciences, Kyushu University, Fukuoka, Japan; 3 Department of Biology, Faculty of Science, Kyushu University, Fukuoka, Japan

**Keywords:** AAA+ family, budding yeast, DNA replication, origin recognition complex, origin specificity, replication initiation, single-stranded DNA, structure–function relationship

## Abstract

Single-stranded DNA (ssDNA) is an essential intermediate of genome duplication but can also arise in the genome, including at highly transcribed loci. Although the origin recognition complex (ORC), a eukaryotic replication initiator, has been reported to bind ssDNA, how interactions with ssDNA-exposing regions are regulated without compromising origin specificity and genome stability remains poorly understood. Here, we characterize the ssDNA-binding properties of budding yeast ORC and the role of an initiator-specific motif (ISM)-related region within the AAA+ domain of Orc1. *In vitro*, ORC binds ssDNA (except for poly dA) at affinities comparable to those of replication protein A (RPA) under identical binding conditions, suggesting base composition-dependent modulation of ORC–ssDNA binding. Genome-wide analyses show partial overlap between ORC- and RPA-enriched peaks, consistent with ORC binding at a subset of ssDNA-forming loci *in vivo*. Mutations of the Orc1 ISM-related region increase ORC binding to ssDNA and promote higher-order ORC–ssDNA complex formation *in vitro*, indicating that this region normally limits ORC–ssDNA binding. In contrast, these mutations impair origin-specific ORC binding through mechanisms involving the essential A-element-proximal region. Genetic and chromatin-based analyses further reveal that enhanced ssDNA binding correlates with reduced origin binding *in vivo*, indicating a redistribution of ORC from replication origins to ssDNA-forming loci. Despite conservation of the ISM across domains of life, these results suggest that eukaryotic ORC has functionally diverged such that origin binding is coupled to the repression of ssDNA binding through the Orc1 ISM-related region, thereby safeguarding faithful origin selection.

## 1 Introduction

**Figure 1:**
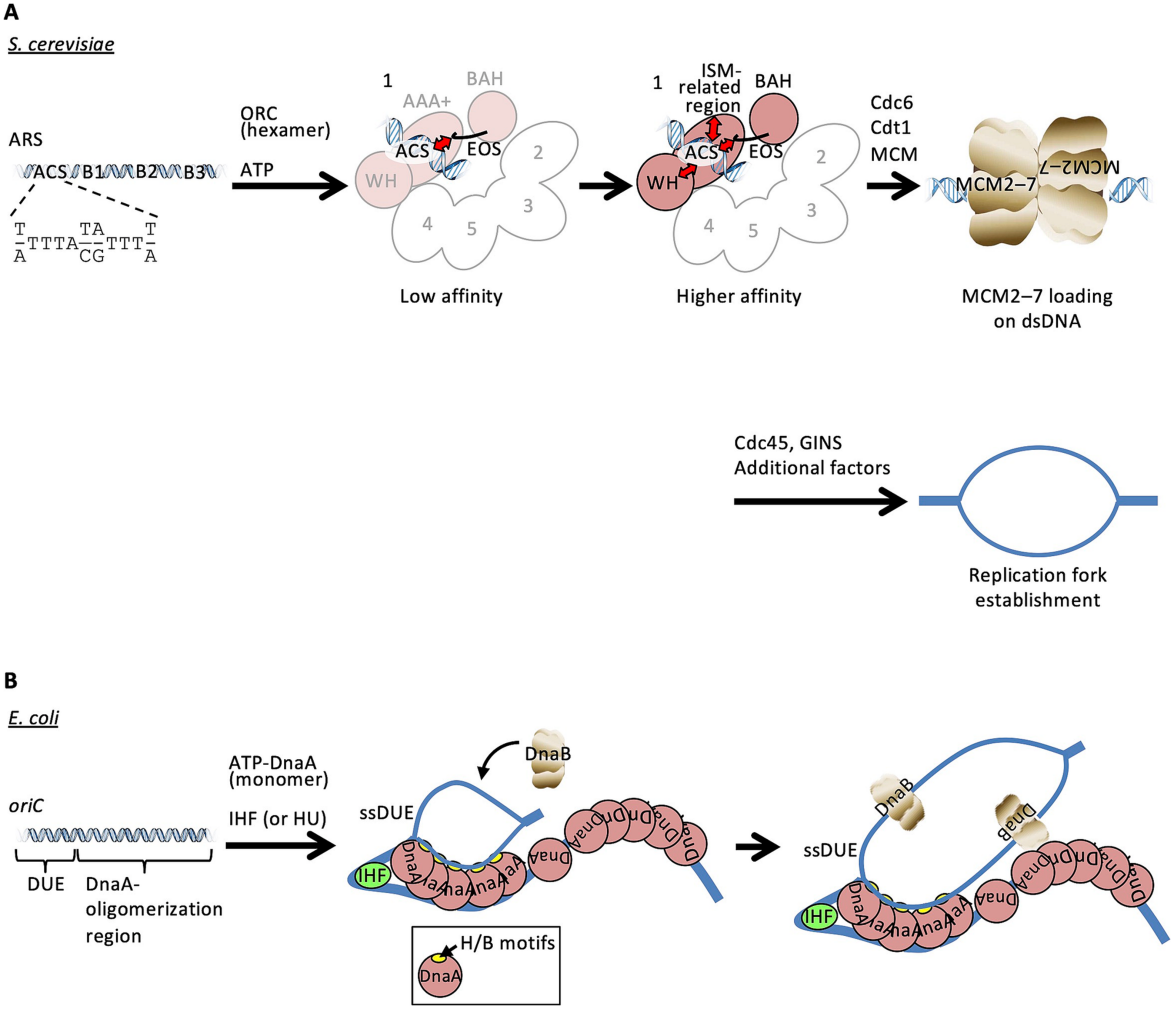
Diversity of replication origin recognition and DNA structural transitions. **(A)** In *S. cerevisiae*, ORC heterohexamer recognizes ARS in an ATP-dependent manner. During this step, the ARS consensus sequence (ACS) is initially recognized with low affinity by the EOS basic patch of Orc1. EOS is located between the bromo-adjacent homology (BAH) and AAA+ ATPase domains of Orc1. Once this recognition is established, ORC is competent to associate with ARS with higher affinity, along with other DNA-binding domains, resulting in loading of MCM2–7 onto dsDNA. Recruitment of Cdc45, GINS, and additional factors promotes activation of the replicative helicase, resulting in local DNA unwinding and establishment of replication forks. Orc1 AAA+ domain containing the ISM-related region and Orc2/3/4/5 are shown in pink and white, respectively. Orc6 and the winged helix (WH) domains of Orc2/3/4/5 are omitted for clarity. **(B)** In *E. coli*, ATP-bound DnaA monomers (pink) assemble into multimers at the DnaA-oligomerization region of *oriC*. DNA bending by IHF (light green) binding induces torsional stress in *oriC* DNA, stimulating DUE unwinding, and the resulting ssDUE is directly bound by specific DnaA protomers and serves as a substrate for DnaB loading.

Eukaryotic DNA replication initiates at specific genomic regions termed replication origins (Kawakami and Katayama, 2010; Parker et al., 2017; Stillman et al., 2025). Accurate recognition and activation of these origins are essential for faithful genome duplication, and defects in this process are associated with genome instability, including chromosomal rearrangements and replication-associated DNA damage. In eukaryotes, replication origin recognition is mediated by the origin recognition complex (ORC), a heterohexameric ATP-dependent DNA-binding complex composed of Orc1–6, which serves as a platform for recruitment of Cdc6, Cdt1, and the MCM2–7 helicase core during M and G1 phases. ORC promotes the formation of DNA-encircling loading intermediates (Figure 1A) (Sun et al., 2012, 2013). The resultant MCM2–7 double hexamer is inactive and later converted into the active replicative helicase, the CMG (Cdc45–MCM2–7–GINS) complex, through phosphorylation-dependent steps. Origin DNA is subsequently unwound during initiation in S phase to form single-stranded DNA (ssDNA), which serves as a template for DNA synthesis.

While ORC is highly conserved among eukaryotes, the genomic features of replication origins exhibit substantial diversity across species (Kawakami and Katayama, 2010; Hu and Stillman, 2023). The budding yeast *Saccharomyces cerevisiae* (Sc) represents a clade in which origin recognition shows a high degree of sequence dependence. Replication origins in *S. cerevisiae* are defined as autonomous replicating sequences (ARSs), originally identified as genomic DNA elements capable of conferring autonomous plasmid replication. ARSs consist of an essential A element and multiple B elements that contribute to replication efficiency (Figure 1A). Both A and B elements share homology with the 11-bp ARS consensus sequence (ACS), with the A element showing perfect conservation. ORC binds to the A element and the adjacent B1 element (Speck et al., 2005), and the A element contributes most strongly to the sequence dependence of ARS function.

Outside *S. cerevisiae* and its close relatives, fission yeast origins exhibit only a preference for AT-rich sequences, and in higher eukaryotes, replication origins generally lack clear sequence specificity (Hu and Stillman, 2023). This indicates that ORC–origin binding involves structures that recognize defined DNA sequences or sequence-correlated regions, particularly in budding yeast, and structures that mediate sequence-independent DNA binding. The former are thought to have diversified during evolution in accordance with species-specific origin properties, whereas the latter are expected to be conserved among eukaryotes. Several species-specific structural elements in ORC have been implicated in sequence-dependent origin recognition, including the N-terminal AT-hook of fission yeast Orc4 and an extra *α*-helix insert within the ATPases associated with a variety of cellular activities (AAA+) domain of ScOrc4 (Lee et al., 2001, 2021). We previously found that ScORC directly recognizes the ARS A element through a basic patch within an intrinsically disordered region (IDR) of Orc1, termed the eukaryotic origin sensor (EOS) motif, which is required for stable ORC–origin binding (Kawakami et al., 2015). EOS-dependent recognition of the A element can occur independently of ATP-binding, although at low affinity, as shown by EMSA with EOS-containing peptides (Kawakami et al., 2015). These findings indicate that ATP is not strictly required for sequence-specific origin recognition *per se* and instead contributes to stabilization of ORC–ARS complexes by structured domains. Binding of ORC to ARS DNA represses ORC ATPase and stabilizes the ATP-bound form of the complex, a state important for efficient MCM2–7 loading (Klemm et al., 1997; Klemm and Bell, 2001). Key EOS residues are conserved among higher eukaryotes, suggesting that this mechanism represents a common basis for origin recognition even in organisms where strong sequence specificity is absent (Kawakami et al., 2015; Bleichert et al., 2018; Li et al., 2018). By contrast, the AAA+ fold shared by Orc1–5 and the adjacent winged-helix (WH) domain are conserved structural features thought to contribute to sequence-independent ORC–dsDNA binding, with the exception of ScOrc4 as noted above. ScOrc1–Orc5 are required for ORC binding to dsARSs, whereas Orc6 is dispensable (Lee and Bell, 1997; Kawakami et al., 2016; Li et al., 2018). The primary ATPase active site resides in Orc1, but efficient ATP hydrolysis requires the conserved arginine finger provided *in trans* by Orc4 (Klemm et al., 1997; Bowers et al., 2004). ATP binding and hydrolysis are therefore coupled to conformational transitions of the ORC complex. Structural analyses in *Drosophila* have shown that ATP binding promotes opening of an autoinhibited ORC conformation permitting DNA entry into the central channel (Bleichert et al., 2018), and ATPase-defective ScORC can support MCM2–7 loading *in vitro* (Coster et al., 2014). However, genetic perturbation of ORC ATPase severely compromises cell viability *in vivo* (Klemm and Bell, 2001; Bowers et al., 2004). These observations indicate that ATP binding and hydrolysis primarily regulate ORC conformational transitions and do not constitute the molecular basis for direct recognition of the A element by the EOS.

In eubacteria, mechanisms for origin duplex unwinding upon initiation have been well characterized (Katayama et al., 2017). In *Escherichia coli*, the eubacterial ORC ortholog DnaA oligomerizes on the replication origin *oriC*, and a nucleoid-associated protein (IHF or HU) induces or stabilizes DNA bending within *oriC*, which promotes duplex unwinding within the DNA unwinding element (DUE) (Figure 1B) (Sakiyama et al., 2017; Yoshida et al., 2023). Stabilization of exposed ssDNA requires specific motifs within the AAA+ domain of DnaA, including the hydrophobic (H) and basic (B) motifs (Ozaki et al., 2008). These motifs are located within and adjacent to the initiator-specific motif (ISM), a helical insertion characteristic of replication initiator/loader AAA+ proteins (Dueber et al., 2007). The H/B-motifs in *E. coli* DnaA directly bind ssDNA to stabilize origin duplex unwinding. Archaeal and eukaryotic ORC complexes also contain regions corresponding to the ISM (hereafter referred to as ISM-related regions) within their AAA+ domains. However, these regions have primarily been shown to cooperate with WH domains in binding double-stranded (ds) DNA (Dueber et al., 2007; Gaudier et al., 2007; Li et al., 2018) instead of binding ssDNA as observed in eubacteria. Although the ISM is conserved across domains of life, its functional contributions appear to have diverged between DnaA and ORC-type replication initiators.

Although ORC-type initiators have diverged from bacterial DnaA in their primary mode of origin binding, eukaryotic ORC retains the ability to bind ssDNA *in vitro* (Lee et al., 2000; Hoshina et al., 2013; Kawakami et al., 2015). We previously reported that ScORC multimerizes on ssDNA in an EOS-dependent manner, stimulating ORC ATPase activity (Kawakami et al., 2019). However, how ORC–ssDNA binding is regulated and how it contributes to ORC function *in vivo* remain unclear. The ScMCM2–7 helicase core is loaded onto dsDNA (Evrin et al., 2009; Remus et al., 2009), raising the question of why ORC retains ssDNA-binding activity. *In vivo*, ssDNA can be transiently exposed at non-origin loci through transcription-associated processes including R-loop formation, which generates a DNA:RNA hybrid with a displaced ssDNA strand (Aguilera and García-Muse, 2012), raising the possibility that ORC encounters ssDNA outside replication origins. This discrepancy suggests that ssDNA binding by ORC may serve regulatory roles distinct from origin unwinding, or may need to be actively restrained to ensure origin specificity *in vivo*. Exposed ssDNA in cells is rapidly coated by the single-stranded DNA binding protein replication protein A (RPA) (Wold, 1997). Therefore, any physiologically relevant ssDNA binding by ORC should be considered as potential competition with RPA. If ORC–ssDNA binding is restricted *in vivo* by competition with RPA, the ISM-related region in ScORC may contribute to origin control directly or indirectly, unlike the H/B motifs of eubacterial DnaA that promote ssDNA stabilization during origin unwinding.

In this study, we examine the ssDNA-binding properties of ScORC and investigate the contribution of an ISM-related region within the AAA+ domain of Orc1. By combining biochemical and genetic experiments with analyses of published genomic datasets, we focus on a conserved ISM-related region in Orc1 and test whether it regulates ssDNA binding analogously or divergently from the eubacterial DnaA. Our results demonstrate that the Orc1 ISM-related region behaves differently from DnaA in regulating origin and ssDNA binding, consistent with the idea that origin binding is coupled to repression of ssDNA binding, thereby safeguarding origin-specific ORC binding *in vivo*.

## 2 Materials and methods

### 2.1 Antibody, DNA, and strains

An anti-Orc1 monoclonal antibody was described previously (Kawakami et al., 2016). Most *ORC1/2/3/4/5/6* plasmids used in this study were derivatives of the yeast shuttle vectors pRS403 (*HIS3*), pRS415 (*ARSH4 CEN6 LEU2*), and pRS416 (*ARSH4 CEN6 URA3*) (Sikorski and Hieter, 1989), as well as the mammalian overexpression vector 3–5 (Uno et al., 2012), as described previously (Kawakami et al., 2015, 2016). pSPB13, a pRS413 (*ARSH4 CEN6 HIS3*) derivative carrying *ORC1*, was a gift from Dr. Bruce Stillman. *orc1* mutations in these plasmids were introduced by site-directed mutagenesis and verified by sequencing. p11d-tRPA was described previously (Henricksen et al., 1994). pGEX/3C, encoding GST-tagged human rhinovirus 3C protease (GST-3C), was a gift from Dr. Arie Geerlof. pHK151, a pGEX-6P-1 (Cytiva)-based vector for GST-Orc1-HisStrepII expression, containing a GST-3C cleavage site between GST and Orc1, was constructed by Gibson assembly and verified by sequencing. Oligonucleotides and yeast strains used in this study are listed in Supplementary Tables 1, 2, respectively.

### 2.2 Proteins

Wild-type and mutant ORCs were expressed in HEK293T cells and purified by affinity and size-exclusion chromatography as described previously (Kawakami et al., 2015, 2016). Human RPA was overexpressed in *E. coli* BL21(DE3) cells harboring p11d-tRPA and purified as described previously (Henricksen et al., 1994). GST-3C protease was expressed in BL21(DE3) cells harboring pCodonPlus (Agilent) and pGEX/3C, affinity-purified using glutathione agarose, and eluted with reduced glutathione. For purification of Orc1-HisStrepII, GST-Orc1-HisStrepII was expressed in BL21(DE3) cells harboring pCodonPlus and pHK151, affinity-purified using glutathione agarose, and cleaved on-column with GST-3C. The resulting Orc1-HisStrepII was further purified by Ni Sepharose.

### 2.3 EMSA

EMSA was performed as described previously with minor modifications (Speck et al., 2005; Kawakami et al., 2015, 2019). Briefly, 290 bp DNA containing *ARS1* (WT or A^−^ B2^−^ B3^−^) was end-labeled with Cy5-ddUTP using terminal transferase. ORC was incubated with the labeled *ARS1* (1.6 nM as a fragment) in the presence of GC-rich competitor (Speck et al., 2005) or poly dI-dC in 5 μL binding buffer (25 mM Hepes-KOH [pH 7.6], 100 mM KGlu, 5 mM Mg(OAc)_2_, 5 mM CaCl_2_, 5 mM DTT, 5% [v/v] glycerol, 0.1% [v/v] Triton X-100, 2 mg/mL BSA, and 1 mM ATP) for 10 min at room temperature. The reaction was analyzed by 4% native PAGE and either a LAS-4010 or Typhoon Trio+ imager (Cytiva), and quantified using ImageJ. To detect ORC–ssDNA complexes, 96-mer DL15 (Lee et al., 2000), HK461, RM05, RM06, HK467, and HK468 were labeled similarly and replaced *ARS1* (1.2 nM as a fragment). To compare ssDNA binding activity of ORC with that of RPA, competitor DNA was excluded.

### 2.4 ATP-binding assay

ATP binding was assayed as described previously (Klemm et al., 1997; Kawakami et al., 2005, 2015). ORC (1.2 or 2.4 pmol) was incubated with [*α*-^32^P]ATP for 5 min at 30 °C in 25 or 50 μL of buffer K [45 mM Hepes–KOH (pH 7.6), 4.5 mM Mg(OAc)_2_, 140 mM KCl, 9% (v/v) glycerol]. The reaction mixture was filtered through a nitrocellulose membrane (Merck Millipore) pre-equilibrated with ice-cold buffer K. The membrane was washed with 5 mL buffer K, dried, and the retained radioactivity was quantified by liquid scintillation. The dissociation constant and stoichiometry of ORC were calculated by Scatchard plots.

### 2.5 ATPase assay

ATPase assays were performed as described previously with some modifications (Klemm et al., 1997; Kawakami et al., 2016). Briefly, a 76-bp segment of wild-type *ARS1* (position 818–893) or a mutant (noACS) was incubated with 0.4 pmol of ORC and 15 μM [α-^32^P]ATP at 25 °C in 10 μL of buffer identical to buffer K except that it contained 200 mM KCl. At 0, 15, 30, 45, and 60 min, 1 μL of the reaction mixture was stopped by adding 0.5 μL of 2% SDS. The resultant radiolabeled ADP was analyzed by polyethyleneimine-cellulose thin layer chromatography and an FLA-9500 imager (Cytiva).

### 2.6 BglII protection assay

ORC–Cy5-*ARS1* complexes were prepared as for EMSA, incubated with 5 units BglII for 30 min at 30 °C, terminated by adding 0.5 μL of buffer containing 4% SDS and 150 mM EDTA, and analyzed by 4% PAGE and a LAS-4010 imager.

### 2.7 Yeast techniques

Yeast media and cell synchronization were as described (Kawakami et al., 2015). When necessary, 0.5 g/L 5-fluoroorotic acid (5-FOA) was supplemented. Flow cytometry of cells stained with SYTOX Green (Thermo Fisher) was performed using a FACSCalibur apparatus (BD Biosciences), collecting 30,000 events per sample, as described previously (Kawakami et al., 2015). Serial dilutions were performed as described previously (Kawakami et al., 2015). Briefly, cells were adjusted to an OD_600_ of 1 and 10-fold serially diluted. Three microliters of each dilution were spotted onto indicated media. ChAP assays were performed as described previously (Kawakami et al., 2015). Briefly, cells were crosslinked with formaldehyde, lysed, and Orc1-His_12_-associated chromatin was isolated using metal-affinity beads. Recovered DNA was quantified by qPCR using locus-specific primers.

### 2.8 Western blotting

Western blotting was performed as described previously (Kawakami et al., 2016). Briefly, proteins were separated by SDS-PAGE, transferred to nitrocellulose membranes, and detected using an Orc1 antibody and HRP-conjugated secondary antibodies. Signals were visualized by chemiluminescence and quantified by densitometry using ImageJ. Cellular Orc1 levels were estimated by comparison with purified Orc1-HisStrepII as a quantitative standard.

### 2.9 Pre-processing of deep sequencing datasets

Publicly available sequencing datasets were processed as described previously (Kawakami et al., 2019) with minor modifications. ScORC ChIP-seq (SRA: SRX526459) (Belsky et al., 2015) and ScRfa1 ChIP-ssSeq datasets (GEO: GSM1272654) (Yu et al., 2014) were adapter-trimmed using Cutadapt v5.2 (Martin, 2011) and aligned to the sacCer3 reference genome using Bowtie2 v2.5.4 (Langmead and Salzberg, 2012) in very-sensitive mode with default reporting settings, retaining a single best alignment per read. Only uniquely mapped primary alignments were retained for the ORC dataset, whereas for the RPA dataset, only properly paired primary alignments were retained, and unaligned reads were discarded. Potential PCR duplicates were removed using Sambamba v1.0.1 (Tarasov et al., 2015). Only primary alignments with mapping quality (MAPQ) ≥1 were retained for downstream analyses. RPGC-normalized coverage tracks were generated using deepTools3 v3.5.6 (Ramírez et al., 2016) in 1-bp bins, using fragment lengths inferred from read pairs without artificial extension. For R-loop identification assisted by nucleases and sequencing (RIAN-seq) datasets (GSM8657421) (Li et al., 2025), raw reads were processed in the same manner. Properly paired primary alignments with MAPQ ≥10 were retained. Potential PCR duplicates were identified using Sambamba and excluded prior to coverage track generation. Coverage tracks were calculated in 1-bp bins and normalized to RPKM as described in the original report, with artificial read extension disabled.

### 2.10 Genomic co-localization and *de novo* motif discovery analyses of ORC and RPA

Peak calling was performed using SICER2 v1.0.3 (Zang et al., 2009) with a window size of 50 bp and a gap size of 50 bp by comparing ChIP samples with the corresponding input controls. Peaks passing a Benjamini–Hochberg (BH)-adjusted false discovery rate (FDR) ≤5% were used for downstream analyses. Peak summits were defined as the genomic positions showing the maximum RPGC-normalized signal within each SICER2 peak region. Genome-wide annotation data were obtained from the *Saccharomyces* Genome Database (Engel et al., 2024), and all regions annotated as “ARS” and “gene” were analyzed after duplicated entries with identical genomic coordinates were removed. Peaks were converted to summit-centered symmetric windows ranging from ±0 to ±500 bp for downstream analyses. Genomic co-localization of ORC and RPA was defined as ≥1 bp overlap between expanded ORC and RPA peak regions. Overlapping peaks were merged into shared clusters to prevent redundant counting of partially overlapping ChIP signals. Non-overlapping peaks were classified as ORC-only and RPA-only regions. For Venn diagram and motif analyses, shared ORC–RPA loci were defined using summit-centered ±150-bp windows. Overlapping windows were merged, and each merged cluster was represented by a ± 150-bp window centered on the cluster midpoint. *De novo* motif discovery in ORC–RPA shared peak sets was performed using HOMER v5.1 (Heinz et al., 2010) with motif lengths of 6–20 bp. ORC-only and RPA-only regions were used as background sequences to control for ChIP-specific biases and local genomic context. Default HOMER parameters were used unless otherwise noted. Independent motif discovery was performed using MEME-ChIP/MEME Suite v5.5.9 (Bailey et al., 2015), specifying motif widths of 6 to 20 bp and using ±250 bp windows to capture potential flanking sequence architecture. Motif similarity between HOMER- and MEME-derived position weight matrices (PWMs) was assessed using Tomtom/MEME Suite. Motif occurrences in selected genomic regions were evaluated using FIMO/MEME Suite with MEME-derived PWMs using default threshold settings.

### 2.11 k-mer–based validation and dinucleotide-preserving permutation testing

To complement PWM-based motif analyses, sequence-level enrichment was independently evaluated using a sequence-pattern-based Fisher’s exact test. This approach quantifies whether the presence of a defined sequence pattern is disproportionately frequent in ORC–RPA co-localized loci compared to background regions, based on the relative counts of motif-positive and motif-negative sequences in each group, and yields a *p*-value for the observed enrichment. For the rank 1 motif, enrichment of the 10-bp GC-rich near-palindromic core sequence was tested using an exact k-mer representation. For the rank 2 motif, enrichment was evaluated using an 18-bp sequence in which only 10 fixed nucleotide positions were specified, and all other positions were treated as N (any nucleotide). Thus, only the defined fixed positions contributed to matching criteria. Each sequence was scored in a binary manner as motif-positive if it contained at least one instance of the query pattern within the analyzed window, and motif-negative otherwise. For mismatch analyses, N positions were excluded from mismatch counting; mismatches were counted only at fixed (non-N) positions, allowing up to 1–2 mismatches relative to the fixed positions. Sequences were classified as positive if at least one qualifying match occurred within the specified window. Enrichment of motif-positive sequences in ORC–RPA co-localized loci relative to background regions was assessed using 2 × 2 contingency tables and two-sided Fisher’s exact tests, following a standard motif enrichment framework (McLeay and Bailey, 2010). To determine whether the observed enrichment could be explained solely by local nucleotide composition, empirical *p*-values were calculated using dinucleotide-preserving permutation testing. For each input sequence, shuffled sequences were generated that preserved dinucleotide frequencies while disrupting higher-order motif structure (Altschul and Erickson, 1985; Jiang et al., 2008). For each permuted dataset, motif-positive counts were recalculated using the same mismatch criteria. The empirical *p*-value was computed as (*r* + 1)/(*n* + 1), where *r* is the number of permuted datasets showing motif-positive counts greater than or equal to the observed motif-positive count, and *n* = 10,000 permutations (North et al., 2002).

### 2.12 Other computational analyses

Secondary structure prediction and sequence alignment of ScOrc1 protein were performed as described (Kawakami et al., 2015). Amino acid sequence logos were generated using WebLogo v2.8.2 (Crooks, 2004). A homology model of ScOrc1 was generated using SWISS-MODEL (Biasini et al., 2014) with the crystal structure of the *Drosophila melanogaster* Orc1 AAA+ and WH domains (PDB: 4XGC) as the template (Bleichert et al., 2015). Structural models were visualized using UCSF Chimera v1.18 (Pettersen et al., 2004).

## 3 Results

### 3.1 ORC binds ssDNA comparably to RPA in a base composition–dependent, Orc6-independent manner

**Figure 2:**
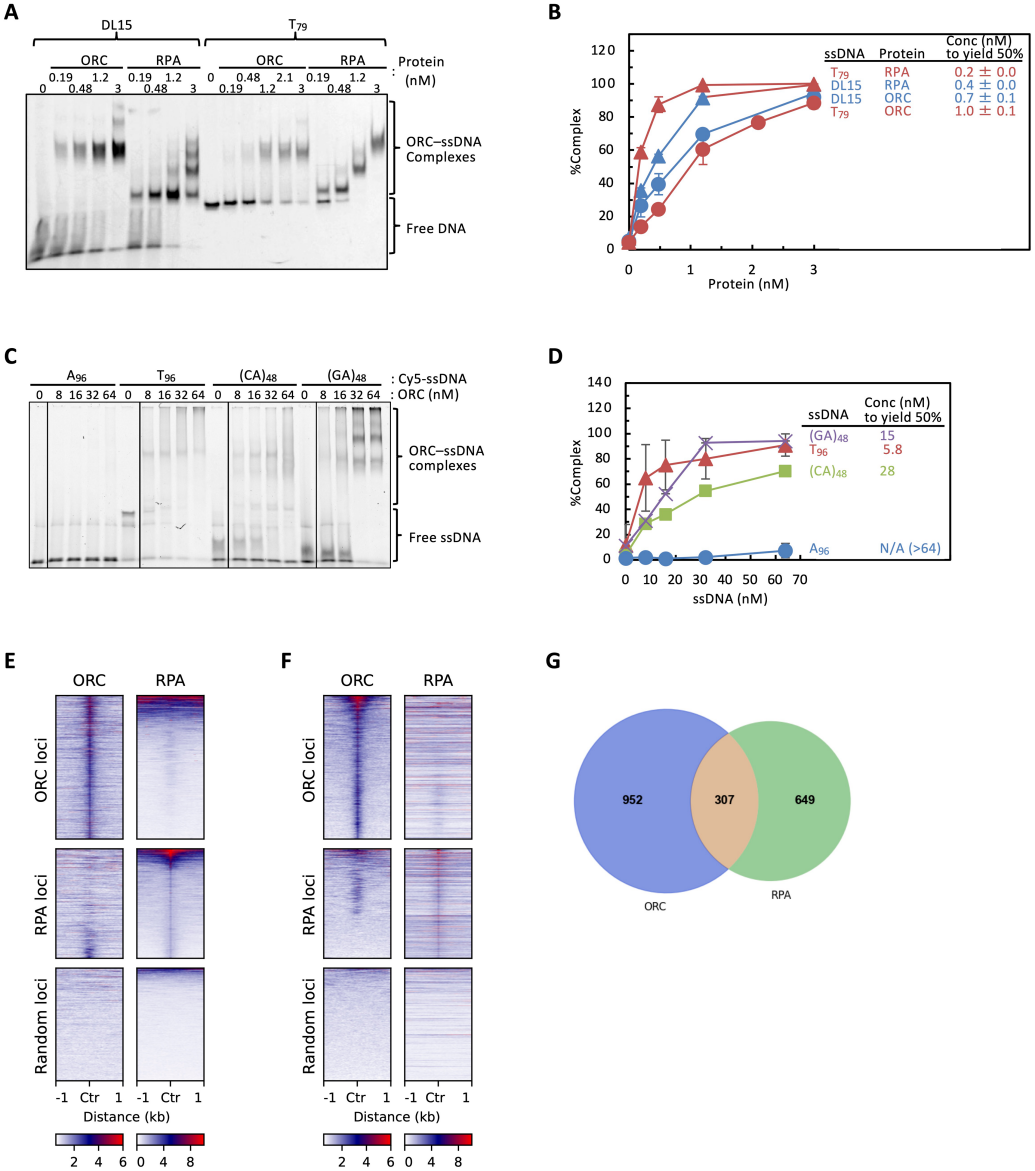
Comparison of ssDNA-binding activities and genome-wide localization of ORC and RPA. **(A)** EMSA of Cy5-labeled DL15 or T_79_ ssDNA with ORC or RPA. Purified ORC and RPA (0–3 nM) were incubated with the indicated ssDNA in the presence of 1 mM ATP and resolved by 4% native PAGE. **(B)** Quantification of panel **A**. The protein concentrations yielding 50% complex formation are indicated. Two independent experiments are shown with mean values. **(C)** EMSA of Cy5-labeled A_96_, T_96_, (CA)_48_, and (GA)_48_ ssDNA with wild-type ORC. All lanes were derived from the same gel. **(D)** Quantification of panel **C**. ORC concentrations yielding 50% complex formation are indicated. Three independent experiments for A_96_ and T_96_ are shown as mean ± SD, whereas (CA)_48_ and (GA)_48_ are shown from a single experiment without error bars. **(E,F)** Heatmaps of ChIP-seq signals for ORC and RPA at ORC-enriched, RPA-enriched, or random loci (±1 kb windows centered on peak summits). All peak calls (BH-adjusted FDR ≤5%) were included to capture overall binding patterns. In panel **E**, regions were sorted in descending order based on the mean RPA signal, whereas in panel **F**, regions were sorted in descending order based on the mean ORC signal. (**G**) Venn diagram showing the overlap between ORC- and RPA-enriched loci using a summit window size of ±150 bp.

Because ssDNA exposed *in vivo* in eukaryotic cells is generally bound by RPA (Chen and Wold, 2014), we first compared ssDNA binding by ORC and RPA under identical conditions. Previous studies have reported that the apparent affinity of ORC for ssDNA is within a few-fold of its affinity for ds ARS DNA and is comparable to that of RPA (Lee et al., 2000). To elucidate factors that influence ORC–ssDNA binding, we first performed EMSA using RPA and the same 96-mer random-sequence probe (DL15) previously used to assess ORC–ssDNA binding (Supplementary Table 1) (Lee et al., 2000; Kawakami et al., 2015, 2019), with excess BSA included to minimize nonspecific binding. RPA bound DL15 with an affinity comparable to that of ORC (Figures 2A,B). A similar result was observed with a 79-mer poly T probe; however, in this case RPA exhibited approximately fivefold higher affinity than ORC (Figures 2A,B), suggesting that ssDNA base composition influences ORC affinity.

To directly examine whether ssDNA base composition influences ORC binding, we compared ORC binding to 96-mer poly dT and poly dA ssDNA probes. ORC bound poly dT robustly but bound poly dA only weakly (Figures 2C,D), indicating that ORC disfavors poly dA sequences. Poly dC and poly dG probes were not analyzed due to unsuccessful end labeling (Supplementary Figure 1). As homopolymeric dC- and dG-rich tracts can form stable non-B-form secondary structures [e.g., i-motif-like or G-quadruplex (G4)-like structures] under certain conditions (Assi et al., 2018; Spiegel et al., 2020), which could reduce DNA end accessibility to terminal transferase, we next tested ORC binding to mixed-sequence probes by introducing C or G into an otherwise poly dA background. Alternating dA with either dC or dG [96-mer (CA)_48_ and (GA)_48_] showed a tendency to partially restore ORC binding (Figures 2C,D), suggesting stronger contributions from dC and dG than dA to ORC–ssDNA affinity. These results demonstrate that ORC–ssDNA binding is sensitive to base composition.

To test whether ssDNA binding occurs via a fundamentally different mechanism from ARS binding or instead shares features with dsDNA binding, we analyzed the ssDNA-binding ability of ORC lacking Orc6 (ORC∆6). We found that ORCΔ6 binds ssDNA with an efficiency comparable to that of wild-type ORC (Supplementary Figure 2). This result indicates that Orc1 through Orc5 are sufficient for ssDNA binding under these conditions and suggests that ssDNA binding, like dsDNA binding, is mediated by the core Orc1–Orc5 subcomplex, all of which contain AAA+ domains.

### 3.2 Genome-wide analysis reveals ORC association with RPA-enriched, ssDNA-forming loci

Given that ORC binds ssDNA *in vitro* in a manner influenced by base composition (Figures 2A–D), we next asked whether ORC could associate with specific ssDNA-forming loci *in vivo* by comparing genome-wide localization profiles of ORC and RPA. Using previously published ChIP-seq datasets in *S. cerevisiae*, we independently obtained peak calls with defined summits for ORC- and RPA-enriched regions (BH-adjusted FDR ≤5%), and visualized signal intensities across peak summits and the surrounding ±1 kb regions as heatmaps. As expected, ORC and RPA signals were enriched at the centers of their respective peak sets (Figures 2E,F). ORC signal was also detected at a subset of genomic regions identified as RPA-enriched, and conversely, RPA signal was observed at a subset of ORC-enriched regions. Such overlaps were not observed when randomly selected genomic loci were analyzed, indicating that this enrichment is not attributable to background signal. To quantitatively assess co-localization between ORC and RPA, we defined summit-centered windows and quantified shared events between the two datasets (Figure 2G). A fraction of ORC and RPA peaks formed shared overlap clusters, while the remaining peaks were condition-specific (ORC-only or RPA-only). The overall overlap trends were consistent across a range of summit window sizes (Supplementary Figure 3). Because ORC and RPA datasets were generated independently, co-enrichment of ORC and RPA in this analysis does not imply stable simultaneous binding of the two factors. These observations are consistent with the possibility that certain genomic loci may temporally unwind *in vivo*, allowing association with ssDNA-binding factors such as ORC or RPA at different times or under different conditions.

### 3.3 Identification of residues analogous to H/B motifs of DnaA in ScORC

**Figure 3:**
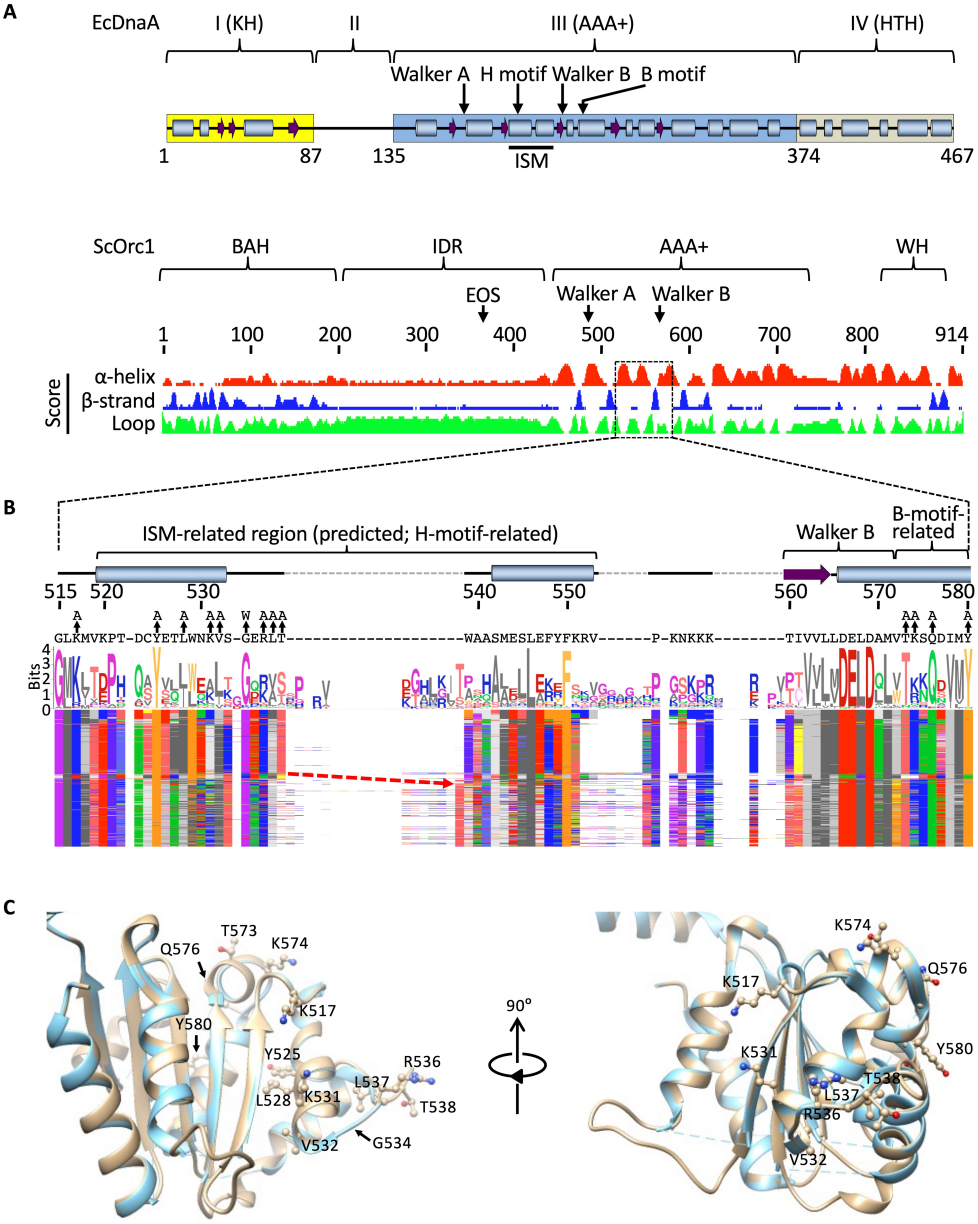
Structural features of Orc1. **(A)** Comparison of *E. coli* DnaA and *Sc*Orc1. For DnaA, see Katayama et al. (2017) for details. For Orc1, the bromo-adjacent homology (BAH), IDR, AAA+, and WH domains as well as EOS and Walker A/B motifs are indicated. Domains and motifs are indicated along with the prediction scores for the secondary structures for each residue. Residues 515–580, corresponding to an ISM-related region initially identified by sequence and structural prediction, are boxed. **(B)** Multiple sequence alignment of the top 500 BLAST hits using *Sc*Orc1 as the query. The residues corresponding to positions 515–580 of *Sc*Orc1 are shown. The sequence logo and residues mutated in this study are shown. The arrow indicates residues aligned to *Sc*Orc1 T538. The variation in conservation across this region reflects species-specific insertions and deletions within the ISM-related segment, resulting in local alignment shifts. See Supplementary Figure 4 for details. **(C)** Homology model of *Sc*Orc1 (gold) overlaid with the crystal structure of the *D. melanogaster* Orc1 AAA+ domain (cyan; PDB: 4XGC). Residues mutated in this study are shown as ball-and-stick models.

To identify Orc1 residues potentially involved in regulating ssDNA binding, we searched for motifs analogous to the H/B motifs of the bacterial initiator DnaA. In the absence of an ORC–ssDNA structural model, this analysis was guided by domain homology and secondary-structure prediction, and by comparison with bacterial DnaA. In DnaA, the H (hydrophobic) motif lies within a conserved helical insertion between the Walker A and B motifs, termed the initiator-specific motif (ISM), and we refer to the analogous Orc1 region as an ISM-related region, whereas the B (basic) motif resides in an adjacent *α*-helix flanking Walker B (Figure 3A). DnaA comprises four major functional domains: an N-terminal protein–protein binding domain, a linker, an AAA+ fold, and a C-terminal helix-turn-helix DNA-binding domain (Katayama et al., 2017). Among the six ORC subunits, only Orc1 shares this four-domain architecture (Figure 3A) (Duncker et al., 2009; Kawakami and Katayama, 2010; On et al., 2018). We therefore focused on Orc1 and identified a helical insertion between the Walker A and B motifs, along with an adjacent α-helix corresponding to the DnaA B-motif (Figure 3B).

Sequence alignment of Orc1 homologs revealed strong conservation across this region, including the conserved acidic residues in the Walker B motif, supporting the reliability of this alignment (Figure 3B; Supplementary Figure 4). Based on the predicted secondary structure and structural positioning, we selected Y525, L528, and V532 as candidates for an H-like motif and K574 as a candidate for a B-like motif. The central loop between the helices is longer in Orc1 than in DnaA (Ozaki et al., 2008), and is highly variable except for residues adjacent to the N-terminal helix (Figures 3A,B; Supplementary Figure 4), suggesting potential functional divergence of this region. We therefore included additional conserved residues as secondary candidates for H/B-like features, identifying K517, K531, G534, and residues R536–T538 as extended candidates for the H-like region, and T573, Q576, and Y580 for additional candidates for the B-like region (Figure 3B). These residues are conserved among representative Orc1 homologs (Supplementary Figure 4).

Mapping these residues onto a homology model of ScOrc1 revealed that most candidate residues are surface-exposed and oriented outward, consistent with their potential roles in DNA binding and/or regulation (Figure 3C). In contrast, Y525 and L528 are oriented toward an internal *β*-strand within the AAA+ fold and may primarily contribute to local structural integrity (Figure 3C, left). Based on these considerations, we constructed the *orc1 R536A L537A T538A* triple mutant, hereafter referred to as *orc1 536-3A*, and used this allele for subsequent biochemical and cellular analyses.

Analysis of available cryo-EM structures further supports this assignment. During origin recognition, sequence-specific recognition of the ACS is mediated by the Orc1 EOS region (K362 and R367) with an extended α-helix in Orc4, whereas residues K520 and W539 contribute to non-sequence-specific contacts with the DNA backbone phosphates (Supplementary Figure 5) (Yuan et al., 2017; Li et al., 2018). W539 is positioned next to Orc1 residues R536–T538. However, consistent with the uncharged nature of tryptophan, the contact between W539 and the DNA backbone phosphate is non-ionic and likely contributes to DNA positioning. K520 and W539 are not conserved among Orc1 homologs (Figure 3B; Supplementary Figure 4).

### 3.4 ATP-binding and ATPase activities of purified ORC containing Orc1 536-3A

**Figure 4:**
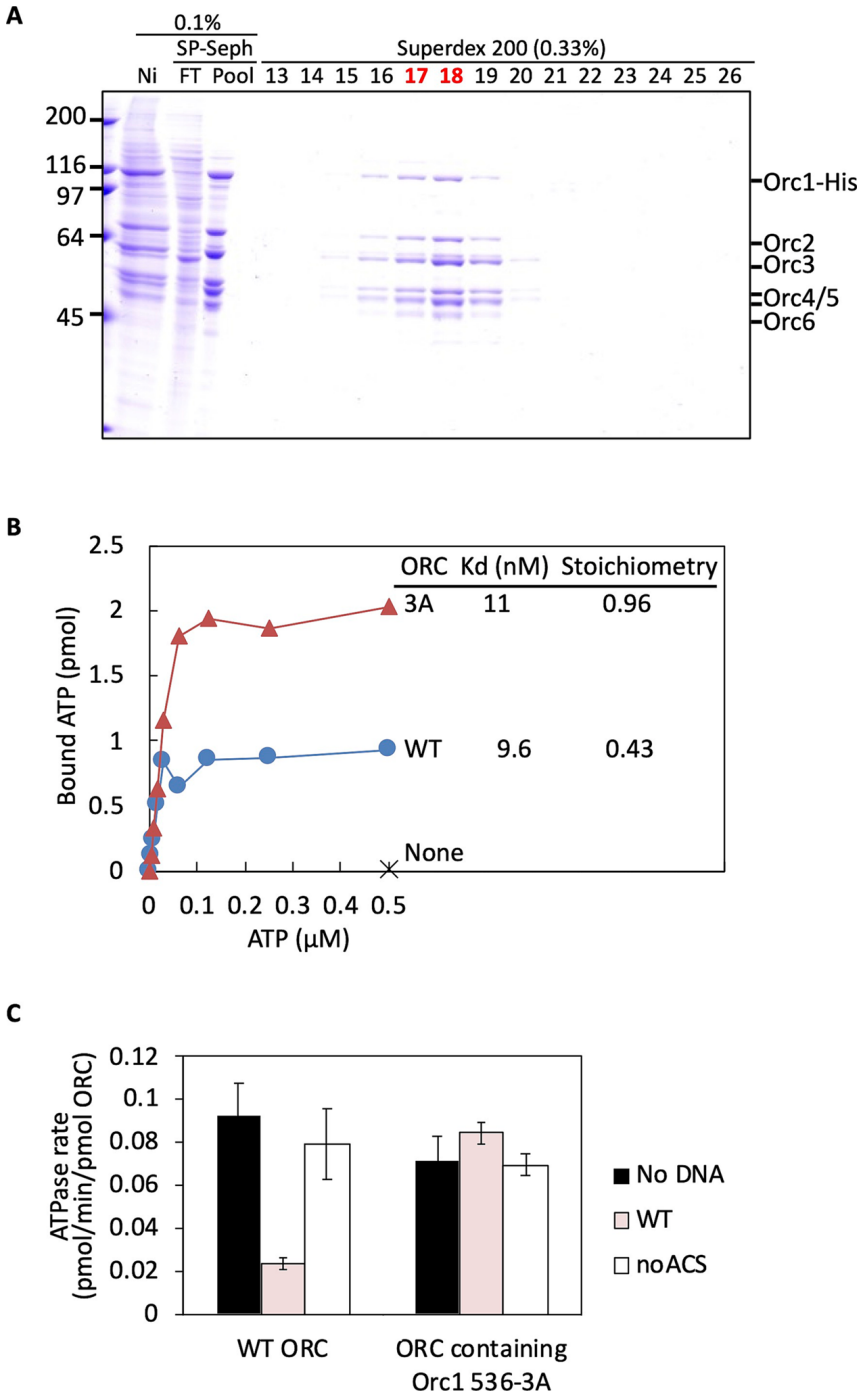
ATP-binding and ATPase activities of ORC containing Orc1 536-3A. **(A)** Purification of ORC containing Orc1 536-3A. HEK293T cells co-overexpressing His-tagged Orc1 536-3A and Orc2–6 were lysed and fractionated. The indicated volumes were analyzed by 9% SDS-PAGE, followed by Coomassie staining. The migration positions of individual ORC subunits are indicated. Ni, Ni-Sepharose pool fractions; FT, flow-through; Pool, pooled fractions; 13–26, fraction numbers. **(B)** ATP-binding affinities determined by nitrocellulose filter-retention assay. Kd and stoichiometry were estimated by Scatchard analysis. See also Supplementary Figure 6A. **(C)** Effects of *ARS1* DNA and the Orc1 ISM-related region on ORC ATPase repression. Wild-type (WT) or mutant (noACS) *ARS1* DNA was incubated with wild-type ORC or ORC containing Orc1 536-3A, and ATPase activity was analyzed over time. Two independent experiments are shown with mean values.

To assess the biochemical activities of Orc1 536-3A within the ORC hexamer, we co-overexpressed Orc1 536-3A with wild-type Orc2–6 subunits in mammalian cells and purified the resulting complex using a previously described streamlined protocol (Kawakami et al., 2016). This procedure, which combines affinity purification via His-tagged Orc1 and gel filtration, yielded an Orc1-containing ORC heterohexamer. The purified mutant ORC was nearly homogeneous, as judged by Coomassie staining, although Orc1 536-3A appeared slightly substoichiometric (Figure 4A and Supplementary Table 3). Minor variation in Orc1 stoichiometry has occasionally been observed in our ORC purifications, including those used for biochemical analysis of the Orc1 EOS mutant (Kawakami et al., 2015, 2019), without affecting downstream assays. Thus, the modest reduction in Orc1 abundance in the 536-3A complex is unlikely to account for the functional differences described below.

To determine whether the Orc1 536-3A mutation affects the core ATP-binding and ATPase activities of ORC, we first analyzed nucleotide binding prior to examining DNA-binding properties. A nitrocellulose filter–binding assay showed that the ATP-binding affinity (Kd) of ORC containing Orc1 536-3A was within the same low-nanomolar range as that of wild-type ORC, whereas the ATP-binding stoichiometry was approximately twofold higher than that of wild-type ORC (Figure 4B; Supplementary Figure 6A). The same trend was observed in an independent assay performed at a different reaction scale (Supplementary Figure 6B). At present, the basis for the increased stoichiometry is not fully defined. One possibility is that the 536-3A mutation alters nucleotide occupancy and/or ATP retention under the filter-binding assay conditions, resulting in a higher stoichiometry without a major shift in affinity. Alternatively, subtle conformational changes in the mutant complex may influence nucleotide accessibility or stability. The Kd and ATP-binding stoichiometry of wild-type ORC were essentially consistent with previous reports, despite differences in expression and purification strategies (Klemm and Bell, 2001; Kawakami et al., 2016). The observed stoichiometry of less than one likely means partial loss of ATP during the assay, as noted earlier (Klemm and Bell, 2001).

ORC ATPase activity is conferred primarily by the Orc1 subunit and is repressed by ds ARS DNA in an Orc1 EOS-dependent manner (Klemm and Bell, 2001; Bowers et al., 2004; Kawakami et al., 2016). In the absence of ARS DNA, ATPase activity of ORC containing Orc1 536-3A was comparable to that of wild-type ORC (Figure 4C, “No DNA”). As expected, ARS DNA significantly repressed ATPase activity of wild-type ORC (Figure 4C). By contrast, this repression was markedly decreased in ORC containing Orc1 536-3A (Figure 4C). These results indicate that the 536-3A mutation does not abolish ATP binding or hydrolysis *per se*, but instead compromises functional ARS-dependent ATPase repression.

### 3.5 Reduced sequence specificity in dsDNA binding of purified ORC containing Orc1 536-3A

**Figure 5:**
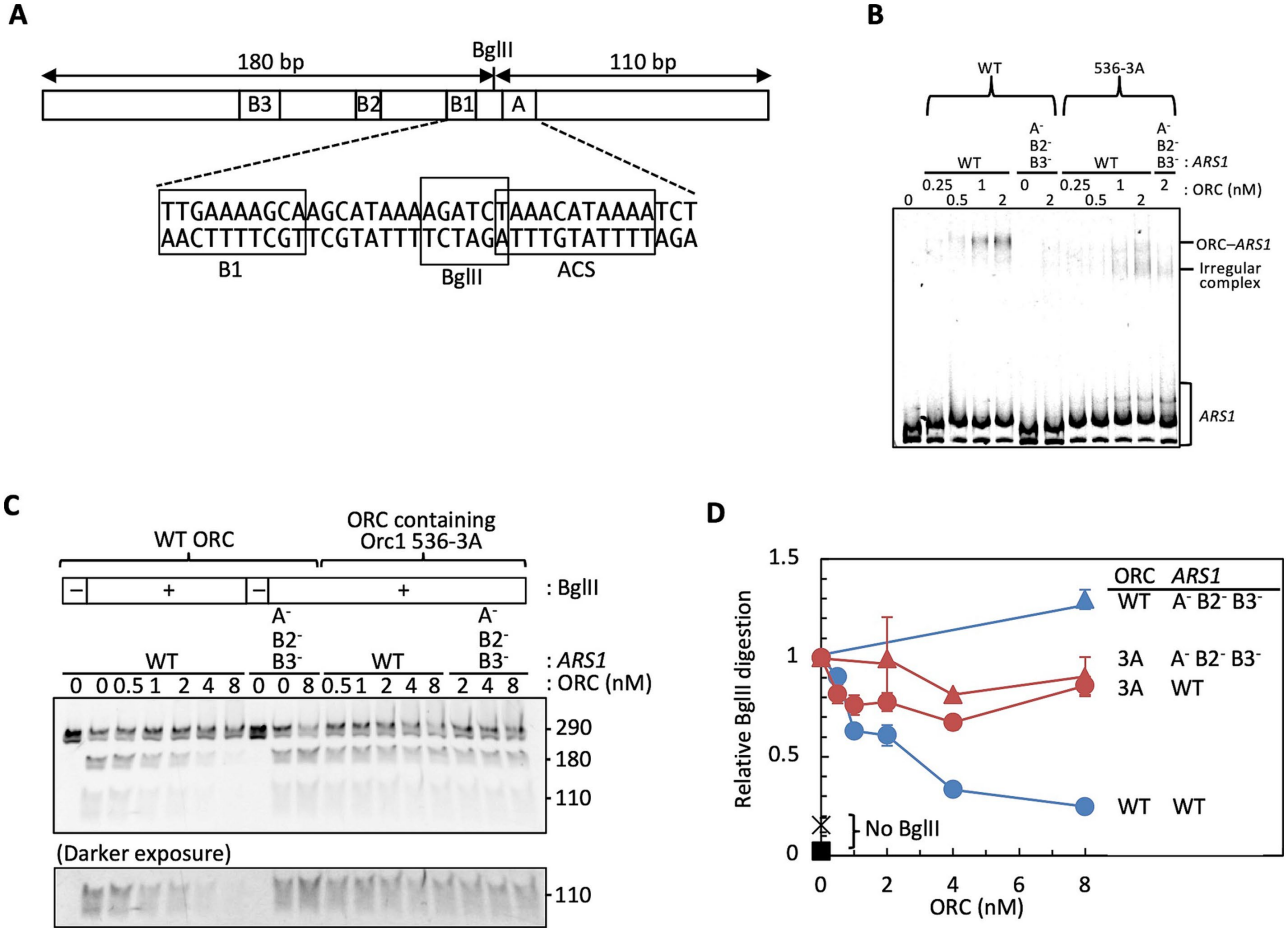
ORC activities in *ARS1* binding and BglII protection. **(A)** Schematic representation of the *ARS1* fragment. The A and B elements, the ACS, and the BglII restriction site are indicated. **(B)** EMSA of Cy5-labeled wild-type (WT) or mutant (A^−^ B2^−^ B3^−^) *ARS1* DNA with wild-type ORC or ORC containing Orc1 536-3A. **(C)** BglII protection assay using ORC and wild-type *ARS1*. Digested DNA fragments were analyzed by 4% PAGE. A darker exposure of the 110 bp bands is shown at the bottom. **(D)** Quantification of the data shown in panel **C**. DNA cleaved by BglII in the absence of ORC was normalized to 1. Two independent experiments were performed, and individual data points and mean values are shown.

To determine how the Orc1 536-3A mutation affects ARS DNA recognition of ORC, we examined *ARS1*-binding activity of the mutant ORC using EMSA (Figures 5A,B). As reported previously, wild-type ORC recognized *ARS1* in a sequence-dependent manner in the presence of competitor DNA (Figure 5B) (Bell and Stillman, 1992; Kawakami et al., 2015). ORC containing Orc1 536-3A also formed complexes with *ARS1* (Figure 5B), but resolved into at least two species: one that migrated similarly to the wild-type ORC–*ARS1* complex (ORC–*ARS1*) and a second, faster-migrating species (hereafter referred to as the irregular complex). The nature of the irregular complex remains unclear, but its formation with both wild-type and mutant *ARS1* fragments (Figure 5B) is more consistent with an altered ORC–DNA binding mode than with reduced complex stability.

To examine this possibility, we next performed competition assays. Both complexes formed by ORC containing Orc1 536-3A were more sensitive to competitor DNA than the wild-type ORC–*ARS1* complex (Supplementary Figure 7). These results suggest that ORC containing Orc1 536-3A retains dsDNA-binding activity with reduced sequence specificity compared with wild-type ORC. At this stage, the reduced sequence specificity may reflect direct consequences of Orc1 residues R536–T538 or those of structural features adjacent to residues R536–T538, including non-sequence-specific backbone contacts via residues K520 and W539 (Supplementary Figure 5) (Yuan et al., 2017; Li et al., 2018).

### 3.6 ORC containing Orc1 536-3A fails to recognize the ACS

The faster migration of the irregular complex relative to the wild-type ORC–*ARS1* complex in EMSA suggested an altered DNA-binding mode, prompting us to examine whether the mutant ORC properly recognizes the ACS sequence. Because ScORC lacking any subunit other than Orc6 fails to bind ARS DNA (Lee and Bell, 1997), the slight substoichiometric abundance of Orc1 in the purified 536-3A ORC fraction (Figure 4A) is unlikely to account for this difference. Instead, altered structural features of the ORC–dsDNA complex provide a more plausible explanation. Bending of linear DNA at its center reduces electrophoretic mobility more than bending near an end (Wu and Crothers, 1984). Given that ORC induces a characteristic bend in ARS DNA (Lee and Bell, 1997) and that ORC containing Orc1 536-3A binds dsDNA with reduced sequence specificity (Supplementary Figure 7), this raised the possibility that the mutant ORC binds at positions other than the A element of *ARS1*, producing faster-migrating complexes than the canonical ORC–*ARS1* complex. Binding at multiple nonspecific positions with heterogeneous bending would be expected to yield a smear in EMSA. However, the irregular complex appeared as a discrete, faster-migrating band (Figure 5B; Supplementary Figure 7A), consistent with a distinct DNA-binding mode involving reduced or altered bending. Although ORC containing Orc1 536-3A may bind near DNA ends, where reduced bending could increase mobility, terminal fraying generates only short-lived ssDNA (Guéron and Leroy, 1995). Since stable ORC–ssDNA complexes generally require longer ssDNA substrates (typically 40-nt or longer) (Lee et al., 2000), it is unlikely that terminal fraying alone produces the distinct, faster-migrating irregular complex observed here.

To test whether the mutant ORC correctly binds the *ARS1* A element, we performed a protection assay using BglII. In this assay, *ARS1* is located near the center of the ~290 bp PCR fragment, and BglII cleaves between the ACS and the B1 element, partially overlapping the ACS (Figure 5A). Because the BglII protection assay measures ORC–DNA interactions in solution prior to electrophoresis, its readout is less affected by electrophoretic stabilization effects that could influence EMSA band patterns. Indeed, wild-type ORC robustly protected this site in an ARS-dependent manner, whereas ORC containing Orc1 536-3A showed little protection (Figures 5C,D). These data indicate that ORC containing Orc1 536-3A fails to protect the ACS-proximal BglII site, consistent with defective ACS binding and/or altered positioning on the *ARS1* fragment. Such altered binding is expected to reduce the characteristic ORC-induced DNA bend, which would increase electrophoretic mobility and could account for the faster-migrating irregular complex. The ds *ARS1* fragment appeared as a doublet in Figure 5C, most likely because incomplete 3′-end labeling with Cy5-ddUTP produced a heterogeneous population of molecules labeled at either one or both 3′ termini, thereby altering electrophoretic mobility (Tu et al., 1998).

### 3.7 Higher ssDNA-binding activity of ORC containing Orc1 536-3A *in vitro*

**Figure 6:**
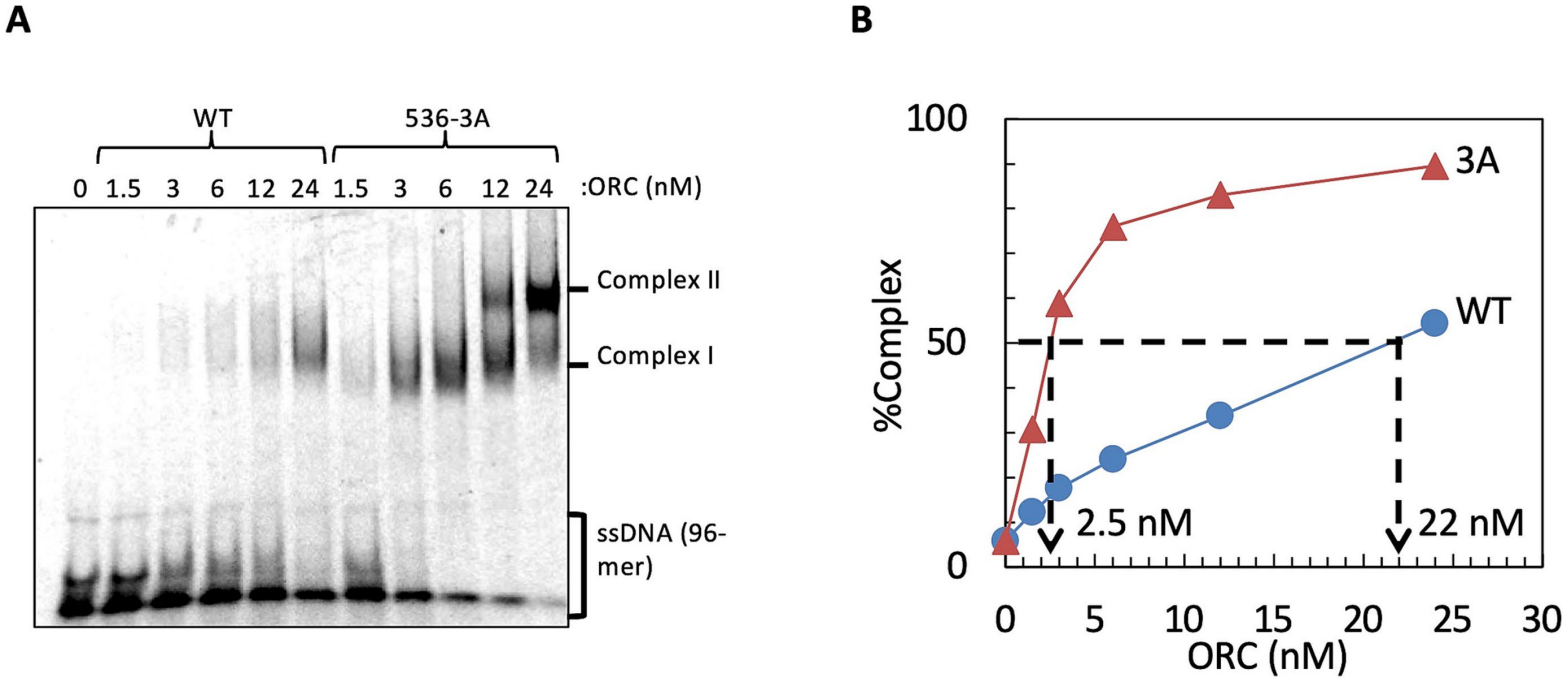
ssDNA-binding activity of ORC containing Orc1 536-3A. **(A)** EMSA of Cy5-labeled ssDNA with wild-type ORC or ORC containing Orc1 536-3A analyzed by 4% native PAGE. **(B)** Quantification of panel **A**.

Because the Orc1 536-3A mutation reduced sequence-specific dsDNA recognition, we next tested whether this alteration was accompanied by changes in ssDNA-binding activity by assessing binding of ORC containing Orc1 536-3A to the DL15 96-mer ssDNA probe. Although the H/B motifs in *E. coli* DnaA are required for ssDNA binding and alanine substitution in these motifs abolishes ssDNA-binding activity (Ozaki et al., 2008), ORC containing Orc1 536-3A bound ssDNA more tightly than the wild-type ORC: the concentration of ORC containing Orc1 536-3A required to shift 50% of the input ssDNA was approximately ninefold lower than that of the wild type (Figure 6). Moreover, higher-order ORC–ssDNA complexes were observed at elevated protein concentrations with the mutant ORC (Figure 6A). This is consistent with previous observations that wild-type ORC also multimerizes on ssDNA at high concentrations in an EOS-dependent manner (Kawakami et al., 2019). These results suggest that Orc1 residues R536–T538 have a role in repressing the ssDNA-binding affinity of wild-type ORC.

### 3.8 *orc1 536-3A* and *orc1 Y525A L528A* are inactive *in vivo*

**Figure 7:**
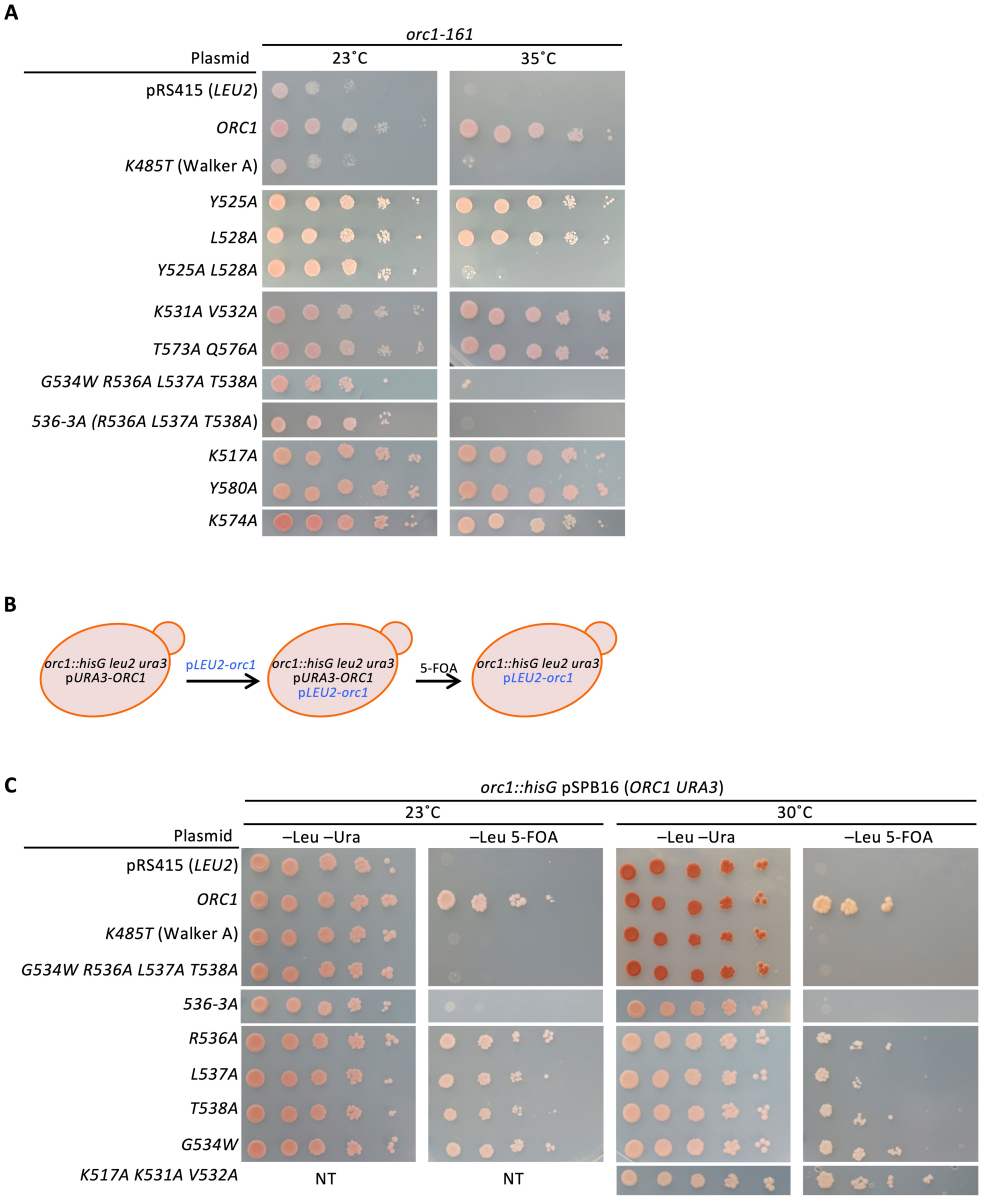
*In vivo* phenotypes of *orc1* 536*-3A*. **(A)** Serial dilution growth assays of OAy66 bearing the indicated *orc1* plasmids at the indicated temperatures. Data from independent experiments were combined. **(B)** Schematic illustration of the plasmid shuffle. In this assay, an *orc1*-disrupted strain carrying a *URA3* plasmid containing wild-type *ORC1* (a derivative of pRS416) was transformed with a low-copy *orc1* plasmid containing *LEU2* (derivatives of pRS415). Cells were then plated on medium containing 5-FOA, which counter-selects cells carrying *URA3* plasmids because the *URA3* gene product converts 5-FOA into the toxic compound 5-fluorouracil. Thus, growth on 5-FOA requires loss of the *URA3* plasmid and the ability of the *orc1* allele on the *LEU2* plasmid to support viability. **(C)** Plasmid shuffle–based serial dilution growth assays of YB838 bearing the indicated *orc1* plasmids at the indicated temperatures. Data from independent experiments were combined. NT, not tested.

To assess the physiological relevance of these biochemical defects, we next examined the *in vivo* functionality of Orc1 mutants using genetic complementation assays. Based on the predicted H/B-like motifs in Orc1 (Figure 3), a total of 13 residues were selected for site-directed mutagenesis and tested in a temperature-sensitive *orc1-161* strain. This strain grows at 23 °C but fails to grow at 35 °C (Kawakami et al., 2015), regardless of the presence of the low-copy centromeric plasmid vector pRS415 bearing an *ARSH4-CEN6* cassette (Sikorski and Hieter, 1989). As expected, expression of wild-type Orc1 from pRS415 restored growth at 35 °C, whereas an allele containing a Walker A mutation did not complement cell growth, consistent with previous reports (Figure 7A) (Kawakami et al., 2015).

We next examined 10 plasmid derivatives carrying single or combined *orc1* mutations. Under these conditions, *orc1 Y525A L528A* and *orc1 G534W R536A L537A T538A*, both located within the ISM-related region, failed to complement the *orc1-161* mutation at 35 °C, whereas *orc1 K517A*, *orc1 K531A V532A*, *orc1 T573A Q576A*, *orc1 K574A*, and *orc1 Y580A* all restored growth (Figure 7A). *Y525A* or *L528A* single mutants restored complementation at 35 °C, suggesting that simultaneous substitution of *Y525A* and *L528A* is responsible for the loss of Orc1 activity. By contrast, a G534 revertant of the *G534W R536A L537A T538A* allele (i.e., *R536A L537A T538A* triple mutant, *536-3A*) did not restore growth, indicating that residues R536–T538 play a central role in Orc1 function. These genetic results obtained with *orc1 536-3A* are consistent with the *in vitro* biochemical phenotypes observed for this mutant (Figures 4–6; Supplementary Figure 7). Y525 and L528 are located in an inward-oriented *α*-helix adjacent to residues R536–L538, and structural analyses indicate that these hydrophobic residues are positioned farther from the DNA than residues R536–L538 (Supplementary Figure 5) (Yuan et al., 2017; Li et al., 2018).

Because these assays were conducted at 35 °C, near the upper limit of the temperature range typically used to assess temperature-sensitive phenotypes, we next examined *orc1* activity at lower temperatures using plasmid shuffling (Figure 7B). Cells receiving the empty vector or an *orc1* Walker A mutant plasmid did not grow at either 23 or 30 °C, whereas the wild-type *ORC1* supported growth, consistent with earlier observations (Figure 7C) (Kawakami et al., 2015). Under these conditions, cells harboring the *orc1 536-3A* plasmid failed to grow at either 23 or 30 °C (Figure 7C), indicating that the *536-3A* allele is not temperature-sensitive and exhibits a defect distinct from the temperature-sensitive phenotype of *orc1-161*. In contrast, single mutants affecting residues R536, L537, or T538 each supported growth (Figure 7C), suggesting that residues R536–T538 function cooperatively or redundantly *in vivo*, and that simultaneous alteration of these residues appears to be required to disrupt Orc1 function. This assay also revealed that K517, K531, and V532 are dispensable for Orc1 function *in vivo* (Figure 7C). These results are consistent with structural analyses (Figure 3C; Supplementary Figure 5) (Yuan et al., 2017; Li et al., 2018).

### 3.9 Orc1 536-3A protein is stable in *Saccharomyces cerevisiae* cells

Because loss of Orc1 function *in vivo* can result from either impaired activity or mutation-dependent protein instability leading to proteolysis, we next examined the cellular abundance of Orc1 mutants. For example, *orc1-161* mutant cells fail to grow at 35 °C owing to a pronounced decrease in Orc1 protein levels (Aparicio et al., 1997; Gibson et al., 2006). To test whether the inactivity of the Orc1 mutants analyzed here can be explained similarly by protein instability, we quantified the expression levels of Orc1 Y525A L528A and Orc1 536-3A by Western blotting.

When wild-type Orc1 was expressed from its native promoter on a low-copy centromeric plasmid in a temperature-sensitive *orc1-161* strain, an Orc1-reactive band was detected at 23 °C and remained at essentially comparable levels at 31–35 °C (Supplementary Figure 8A, lanes 2, 5, 8 and 11). By contrast, endogenous Orc1-161 was already present at low levels at 23 °C and was further reduced in a temperature-dependent manner, consistent with previous results (Aparicio et al., 1997; Gibson et al., 2006) (Supplementary Figure 8A, lanes 1, 4, 7 and 10). Under the same conditions, Orc1 Y525A L528A accumulated to approximately 50% of wild-type levels at 23 °C and decreased further at 31–35 °C (Supplementary Figure 8A, lanes 3, 6, 9 and 12), suggesting that the Y525A and L528A substitutions destabilize Orc1 *in vivo*. Because both residues are hydrophobic and oriented toward the interior of the AAA+ core (Figure 3C; Supplementary Figure 5), we speculate that alanine substitution at these positions disrupts local packing interactions and compromises overall structural integrity.

We next assessed the cellular abundance of Orc1 536-3A. In contrast to Orc1 Y525A L528A, the Orc1 536-3A protein was present at levels comparable to wild-type Orc1 at both 23 °C and 35 °C, even under conditions in which Orc1 Y525A L528A was substantially degraded (Supplementary Figure 8B). We therefore conclude that Orc1 536-3A is stable in budding yeast cells, and that its *in vivo* inactivity (Figure 7) is not attributable to temperature-dependent protein degradation. In contrast, given the instability of the Y525A L528A mutant protein, functional characterization of this mutant would be difficult to interpret independently of its reduced protein abundance. This consideration motivated our focus on the structurally stable *536-3A* mutant for detailed *in vivo* analyses.

### 3.10 Orc1 536-3A delays cell cycle progression during the G1/S and G2/M phases

**Figure 8:**
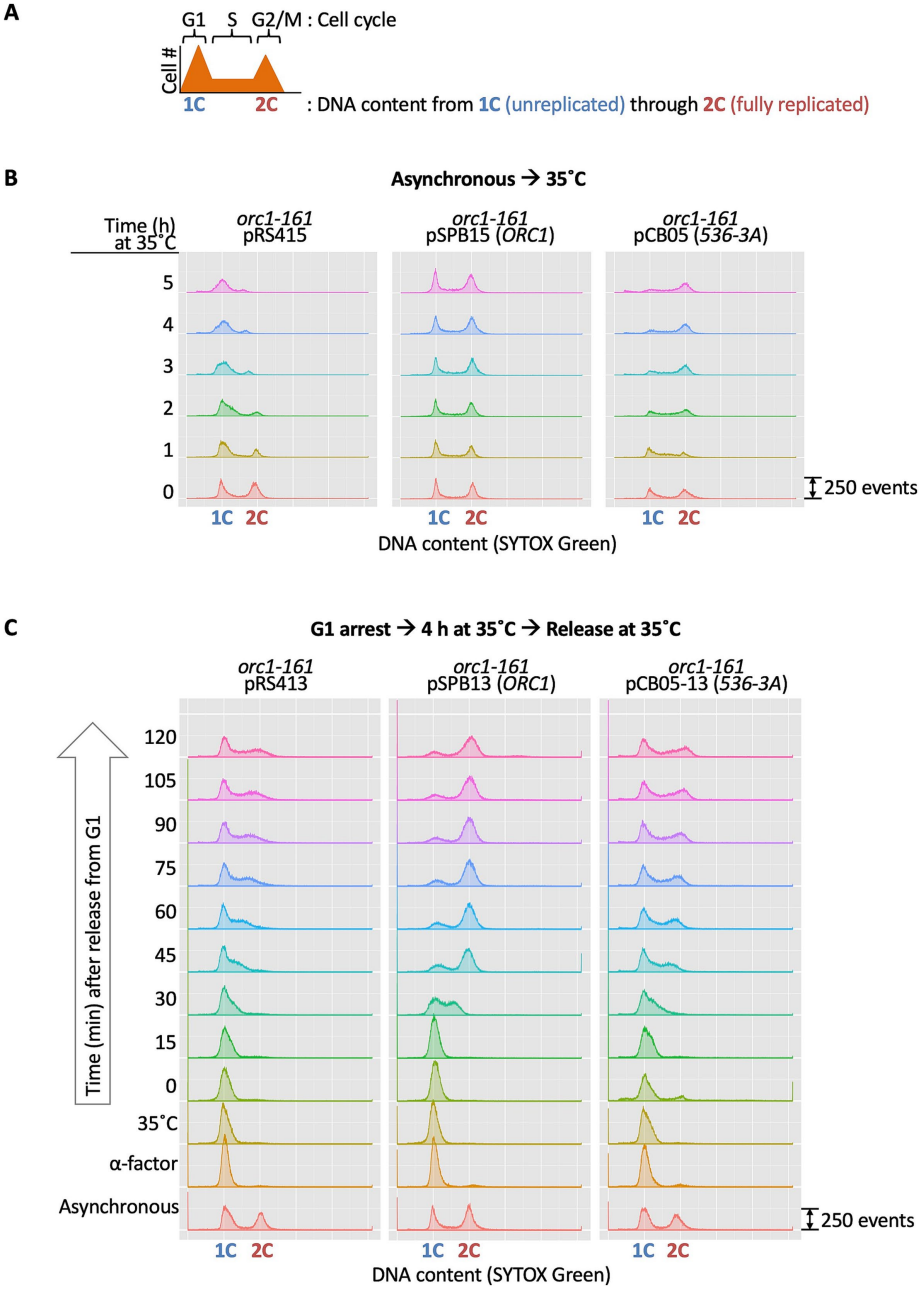
Flow cytometry analyses. **(A)** Schematic illustration of a typical FACS profile. DNA content of unreplicated (1C) and fully replicated (2C) cells is indicated, along with the positions of G1, S, and G2/M phase cells. **(B)** Flow cytometry analysis after temperature shift of asynchronously growing YCB415, YCBB15, and YCB05 cells. Cells were grown at 23 °C to early log phase and shifted to 35 °C. **(C)** Flow cytometry analysis after G1 arrest and release of YCB20, YCB21, and YCB22 cells. Cells were grown at 23 °C, arrested in G1 phase, shifted to 35 °C, and released at 35 °C.

Given the essential role of Orc1 in cell cycle progression, we next investigated how the *orc1* 536-3A mutation affects cell cycle dynamics by FACS analysis (Figure 8). Although the *orc1 536-3A* mutant obtained by plasmid shuffling exhibited a pronounced growth defect (Figure 7C), an *orc1-161* strain harboring an *orc1 536-3A* plasmid was conditionally lethal owing to the thermolabile nature of Orc1-161 (Aparicio et al., 1997; Gibson et al., 2006) (Supplementary Figure 8). This genetic background therefore allowed mutation-specific phenotypes of *orc1 536-3A* to be examined by FACS analysis under non-permissive conditions (Kawakami et al., 2015). When asynchronous cultures were shifted from 23 °C to 35 °C, *orc1-161* cells carrying a wild-type *ORC1* plasmid progressed through the cell cycle normally (Figure 8B). In contrast, at 35 °C, *orc1-161* cells carrying the *orc1 536-3A* plasmid exhibited a modest accumulation of S phase cells at 1 h and a pronounced accumulation of cells with 2C DNA after 3–5 h (Figure 8B). This phenotype is consistent with earlier observations that mutations in *orc1* activate DNA damage and spindle checkpoint pathways (Gibson et al., 2006).

To more sensitively assess the impact of the *536-3A* mutation on DNA replication initiation, cells were synchronized in G1 by treatment with the yeast pheromone *α*-factor at 23 °C. Arrested cells were then shifted to 35 °C to inactivate endogenous *orc1-161* and transferred to fresh medium lacking α-factor at 35 °C. Under these conditions, synchronized cells resume cell cycle progression, and functional Orc1 activity is provided by the plasmid-borne allele. *orc1-161* cells carrying the wild-type *ORC1* plasmid entered S phase approximately 30 min after release and reached G2/M by 45 min (Figure 8C). By contrast, cells carrying the *orc1 536-3A* plasmid or the empty vector exhibited severe delays at G1 exit and/or during S phase, indicating a significant impairment in the initiation of DNA replication. Therefore, the *orc1 536-3A* mutation delays cell cycle progression at the G1/S transition, primarily due to loss of Orc1 initiator function. The accumulation of cells with 2C DNA is likely an indirect consequence of replication defects, although we cannot exclude the possibility that Orc1 536-3A directly perturbs G2/M-associated processes, such as ORC-dependent sister-chromatid cohesion, as proposed previously (Shimada and Gasser, 2007).

### 3.11 Orc1 536-3A is defective in ARS binding *in vivo*

**Figure 9:**
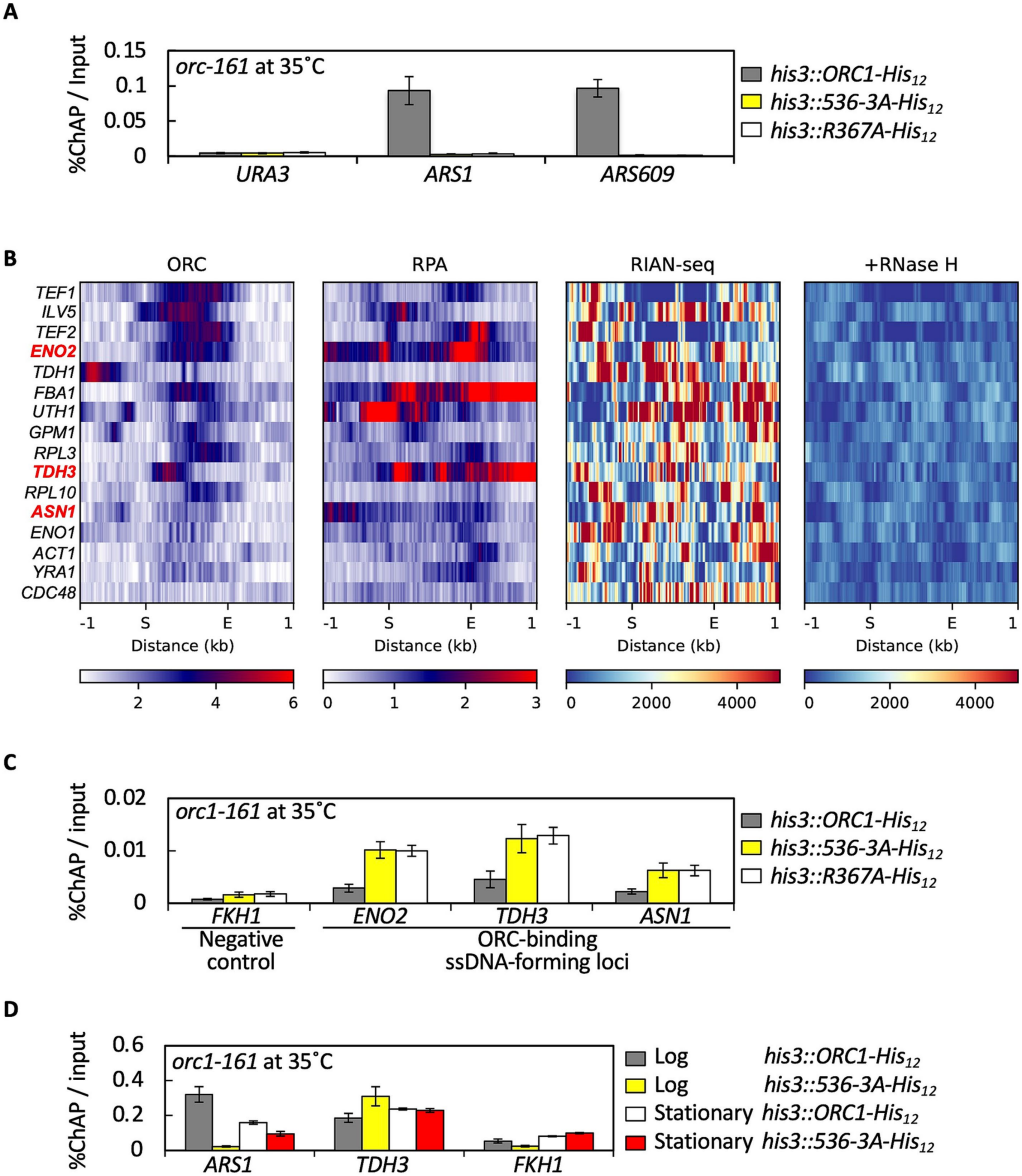
Orc1 residues R536–T538 regulate ORC redistribution between replication origins and ssDNA-forming loci *in vivo*. **(A)** ChAP analysis of chromosomal representative ARSs crosslinked to wild-type or mutant Orc1-His_12_ in YHK33, YCB3A, and YHK35 cells (means ± SE, *n* = 3). **(B)** Genome-wide ChIP-seq and R-loop signal profiles at Shor’s reported ORC-binding loci. Heatmaps of ORC, RPA, RIAN-seq, RNase H-treated RIAN-seq signals at genes previously reported as ACS-independent ORC-binding loci by Shor et al. (2009) (*n* = 16). For visualization, annotated regions were scaled to a uniform length and plotted from the start (S) to the end (E). Regions were sorted in descending order based on the mean ORC signal. Individual genes are shown, and selected genes analyzed in panel **C** are shown in bold red. **(C)** ChAP analysis of Shor’s reported ORC-binding loci crosslinked to wild-type or mutant Orc1-His_12_ in YHK33, YCB3A, and YHK35 cells (means ± SE, *n* = 3). **(D)** ChAP signals of YHK33 and YCB3A cells were compared in log and stationary phases (means ± SE, *n* = 2).

Given the severe cell cycle defects (Figure 8) and reduced ARS selectivity of ORC containing Orc1 536-3A observed *in vitro* (Supplementary Figure 7), we tested whether ORC containing Orc1 536-3A also associates inefficiently at replication origins *in vivo* (Figure 9A). To address this, we performed chromatin affinity purification (ChAP), a modified chromatin immunoprecipitation (ChIP) method in which His_12_-tagged Orc1 is isolated by metal-affinity pulldown under denaturing conditions from a temperature-sensitive *orc1-161* strain incubated at non-permissive temperature (35 °C) (Kawakami et al., 2015). This approach circumvents the underestimation of ORC occupancy at ARSs that can occur in conventional ChIP assays under native conditions due to occlusion by origin-bound replication proteins during G1 phase (Aparicio et al., 1997). We previously showed that His_12_-tagged Orc1 expressed from *his3:ORC1-His_12_* allele suppresses the temperature-sensitive phenotype of *orc1-161* cells and binds representative ARSs in an EOS-dependent manner *in vivo* (Kawakami et al., 2015). Consistent with this, wild-type Orc1-His_12_ was enriched at *ARS1* and *ARS609*, but not at the non-origin control *URA3* (Figure 9A). As reported previously, Orc1 R367A-His_12_, which carries a mutation in the EOS motif, showed no significant association with these ARSs (Figure 9A) (Kawakami et al., 2015). Under the same conditions, Orc1 536-3A-His_12_ failed to associate detectably with either *ARS1* or *ARS609* and showed no enrichment at *URA3*. These results suggest that the 536-3A mutation disrupts a function of Orc1 critical for ARS binding *in vivo*, partially phenocopying the EOS-defective *orc1 R367A* mutant, although the distinct roles of residues R367 and R536–T538 remain to be elucidated.

### 3.12 Orc1 536-3A exhibits increased association with ssDNA-forming loci *in vivo*

Given the enhanced ssDNA-binding activity of ORC containing Orc1 536-3A observed *in vitro* (Figure 6), we next examined whether this increased ssDNA binding is associated with altered ORC distribution *in vivo*. We previously reported that ORC–ssDNA binding can occur genome-wide outside canonical ARSs, including highly transcribed genes, in some cases in association with R-loop formation (Kawakami et al., 2019). Consistently, genome-wide analyses revealed partial overlap between ORC and RPA-enriched loci (Figures 2E–G). Based on these observations, we selected well-characterized loci such as *ENO2*, *TDH3*, and *ASN1*, at which transcription promotes ORC binding in an ACS-independent manner, originally identified by ORC ChIP-chip (Shor et al., 2009). Reanalysis of publicly available ORC ChIP-seq datasets verified ORC binding to these loci (Figure 9B, ORC). Further reanalysis suggested that Shor’s ORC loci, including these three genes, exhibit detectable RPA binding and R-loop signals (Figure 9B; Supplementary Figure 9), supporting the idea that ssDNA exposure may occur at these sites. This motivated us to examine Orc1 association using ChAP-qPCR in a temperature-sensitive *orc1-161* mutant at the non-permissive temperature (35 °C). Note that *FKH1* was used as the negative control instead of *URA3*, because ACS-independent ORC binding is expected to be weaker than *bona fide* ARS binding, and the strain background used for ChAP experiments carries two copies of the *URA3* locus (Supplementary Table 2), which could elevate background signals and obscure weak ORC enrichment. Notably, Shor et al. (2009) identified ORC peaks within highly transcribed genes, including *ENO2*, *TDH3*, and *ASN1,* that lacked functional ACS motifs and did not function as replication origins; these ORF-associated ORC sites were also *orc2-1*(ts)-resistant binding sites, supporting the idea that they represent a binding mode distinct from canonical origin recognition. Indeed, wild-type Orc1-His_12_ was enriched at *ENO2*, *TDH3*, and *ASN1* relative to the negative control *FKH1*, consistent with previous ChIP-based observations (Figure 9C) (Shor et al., 2009). Under the same conditions, Orc1 536-3A-His_12_ exhibited approximately 2–4-fold higher enrichment at these loci than wild-type Orc1 (Figure 9C), in agreement with the enhanced ssDNA-binding activity observed *in vitro* for ORC containing Orc1 536-3A (Figure 6). Transcription-associated DNA structures such as R-loops, which generate DNA:RNA hybrids with displaced single-stranded DNA, are known to form at highly transcribed loci *in vivo* (Aguilera and García-Muse, 2012). The increased association of Orc1 536-3A with these loci is therefore consistent with enhanced ORC binding to ssDNA-exposing regions.

### 3.13 Redistribution of ORC to ssDNA-forming loci upon loss of origin binding

Given the enhanced ssDNA-binding activity of ORC containing Orc1 536-3A observed *in vitro* (Figure 6), we next addressed the mechanisms underlying its increased association with ssDNA-forming loci *in vivo* (Figure 9C). To distinguish between direct and indirect contributions to the increased association, we considered two non-mutually exclusive mechanisms. One possibility is a direct effect, in which the elevated intrinsic ssDNA-binding activity of ORC containing Orc1 536-3A promotes its association with ssDNA-exposing genomic regions. Alternatively, an indirect mechanism may also contribute: because ORC containing Orc1 536-3A fails to associate efficiently with replication origins, a larger fraction of ORC may remain unbound to ARSs, thereby increasing the pool of ORC available for redistribution to non-origin genomic regions, including ssDNA-forming loci. To evaluate this possibility, we also examined the chromatin association of an Orc1 EOS mutant (R367A) by ChAP assay, which is defective in representative ARS binding *in vivo* but retains ssDNA-binding activity *in vitro*, although it fails to undergo EOS-dependent ORC multimerization on ssDNA (Kawakami et al., 2015, 2019). Notably, Orc1 R367A-His_12_ also exhibited increased association with ssDNA-forming loci compared with wild-type Orc1-His_12_ (Figure 9C). These results indicate that loss of origin binding by Orc1 contributes to redistribution of ORC to ssDNA-forming loci *in vivo*, supporting a model in which ORC molecules that fail to bind replication origins and/or exhibit enhanced ssDNA-binding activity are redirected to non-origin genomic regions, including loci associated with ssDNA exposure. ORC containing Orc1 536-3A exhibits increased affinity for ssDNA (Figure 6), suggesting that impaired origin association and elevated ssDNA-binding capacity may cooperatively promote redistribution *in vivo*.

To further investigate the determinants of ORC binding to ssDNA-forming loci, we examined whether the binding of Orc1 536-3A is influenced by cellular transcriptional state. In stationary phase cells, in which global transcriptional activity is substantially reduced (Choder, 1991), the enhanced association of Orc1 536-3A with the *TDH3* locus observed under logarithmic growth conditions was no longer evident; instead, Orc1 536-3A binding to *TDH3* was comparable to that of wild-type Orc1 (Figure 9D). In parallel, enrichment of wild-type Orc1 at *ARS1* relative to the negative control *FKH1* was essentially reproduced under these conditions. These results indicate that the increased association of Orc1 536-3A with non-origin loci is transcription-dependent and is diminished when transcription-associated DNA structures are reduced. Consistent with this interpretation, the distinction between canonical ARSs and ssDNA-forming loci observed during logarithmic growth (Figures 9A,C) was diminished in stationary phase cells, in which the elevated association of Orc1 536-3A with *TDH3* was no longer observed and instead became comparable to that of wild-type Orc1, which was accompanied by a detectable association with ARSs above background levels (Figure 9D). These observations support the idea that ORC occupancy at canonical replication origins versus ssDNA-forming loci is dynamically regulated by cellular state, potentially through transcription-associated DNA structures that expose ssDNA *in vivo*.

### 3.14 Genome-wide classification of ORC–RPA co-localized loci

Consistent with the locus-specific analyses presented above (Figure 9), ORC and RPA signals at Shor’s reported ORC loci were generally weaker than those observed at canonical ARSs (Supplementary Figures 9A,C). In addition to these locus-specific analyses, we sought to identify, at a genome-wide scale, genomic regions where ORC co-localizes with RPA and to characterize their shared sequence features. To identify sequence features associated with ORC–RPA co-localized loci, we performed *de novo* motif discovery using HOMER. Two motifs enriched in >50% of target sequences were identified and were enriched relative to ORC-only or RPA-only regions (Supplementary Figure 10A). In the ORC-only control peak set, the top motif identified corresponded to ACS-like sequences (11% of targets vs. 1.1% background), consistent with canonical ORC–ARS binding. In the RPA-only control peak set, the top three motifs identified were G-rich, mixed, and AT-rich (~5% of targets vs. ~0.1% background), indicating that RPA-bound regions are heterogeneous and differ from those associated with ORC–RPA co-localized loci. Thus, ORC–RPA co-localized loci represent a specific subset of genomic regions with distinct sequence features and are not explained by general properties of ORC- or RPA-binding sites.

The most enriched motif among ORC–RPA co-localized loci (rank 1; HOMER P ≈ 10^−98^) contained a GC-rich near-palindromic core sequence (5′-GGTTCGAACC-3′). Independent motif discovery using MEME-ChIP identified a highly similar motif, and Tomtom analysis confirmed a unique best match between HOMER and MEME-derived PWMs (*q* = 2.17 × 10^−8^), indicating that this motif is not algorithm-specific (Supplementary Figure 10B). To quantify enrichment at the sequence level, we performed a contingency table–based analysis comparing ORC–RPA co-localized loci to ORC-only and RPA-only control regions (Supplementary Table 4). Allowing one mismatch, the 10-bp core motif was detected in 20% of ORC–RPA loci but in only 1.5% of control regions (odds ratio ≈ 17; Fisher’s exact test, *p* = 3.2 × 10^−16^). Allowing two mismatches increased detection to 53% versus 9% (odds ratio ≈ 11; *p* = 1.5 × 10^−15^). Dinucleotide-preserving sequence shuffling (10,000 permutations) failed to reproduce the observed enrichment (empirical *p* < 1 × 10^−4^), indicating that the rank 1 motif reflects a specific sequence module rather than GC bias. The rank 1 motif represents a statistically robust sequence feature of ORC–RPA co-localized loci and raises the possibility that such sequences may transiently adopt non-B DNA conformations or locally facilitate ssDNA exposure, potentially contributing to ORC–RPA localization.

A second motif among ORC–RPA co-localized loci (rank 2; HOMER P ≈ 10^−91^) was also identified and independently rediscovered by MEME-ChIP (Supplementary Figures 10A,C). Tomtom analysis confirmed concordance between the HOMER and MEME-derived PWMs (offset = 2; *q* = 6.8 × 10^−9^), indicating reproducibility at the PWM level. Sequence-level analysis revealed significant enrichment relative to control regions (odds ratio ≈ 5–20 depending on mismatch allowance; Fisher’s exact test *p* ≤ 10^−7^; Supplementary Table 4). Although the magnitude of enrichment varied depending on mismatch allowance and in some cases approached that observed for rank 1, the enrichment pattern was less uniformly strong across mismatch conditions. Similar enrichment was observed for reverse-complement matches. Thus, while rank 2 represents a statistically significant sequence feature of ORC–RPA loci, its enrichment is more moderate and may reflect a secondary sequence preference.

Sequences resembling these motifs were observed among ORC sites reported by Shor et al. (2009), but they did not reach statistical significance in our enrichment analyses (Supplementary Table 5). Consistent with this, heatmap analysis showed limited ORC–RPA co-localization at ARSs and across the majority of genes including Shor’s reported loci (Supplementary Figure 9). Because our analysis was restricted to ChIP peak call–positive regions, the loci described here represent a statistically defined subset of ORC–RPA co-localized sites. These findings do not exclude the possibility of additional low-affinity ORC binding at other genomic regions, including gene bodies (Figures 9C,D), but instead define a distinct, motif-enriched class of ORC–RPA co-localized loci characterized by shared sequence features.

## 4 Discussion

In this study, we identify Orc1 residues R536–T538 within the ISM-related region as a previously unrecognized regulatory element that links repression of ORC–ssDNA binding with origin-specific dsDNA binding *in vitro* and *in vivo* (Figures 5–9; Supplementary Figure 7). Biochemical and cellular analyses reveal that mutating this region enhances ORC binding to ssDNA and drives redistribution of ORC from replication origins to ssDNA-forming loci *in vivo*, uncovering a regulatory mechanism by which ORC represses ssDNA binding to ensure faithful origin selection.

A key issue raised by these results is how Orc1 residues R536–T538 can strongly influence origin recognition despite appearing largely distal to dsDNA in available ORC–origin structures (Supplementary Figure 5) (Yuan et al., 2017; Li et al., 2018). Importantly, the ISM/ISM-related element has been implicated in origin recognition in archaeal Orc1 proteins, where this helical insertion can approach origin DNA in distinct conformational states (Dueber et al., 2007; Gaudier et al., 2007). Moreover, even in eukaryotic ORC–origin assemblies, residues adjacent to R536–T538 (e.g., Orc1 W539) lie near the DNA backbone (Supplementary Figure 5), suggesting that modest rearrangements could alter local DNA positioning and/or coupling to sequence-specific recognition motifs. These observations suggest that R536–T538 contribute to origin specificity primarily through regulatory mechanisms that may involve conformational or dynamic effects not necessarily captured by a single structural snapshot.

**Figure 10:**
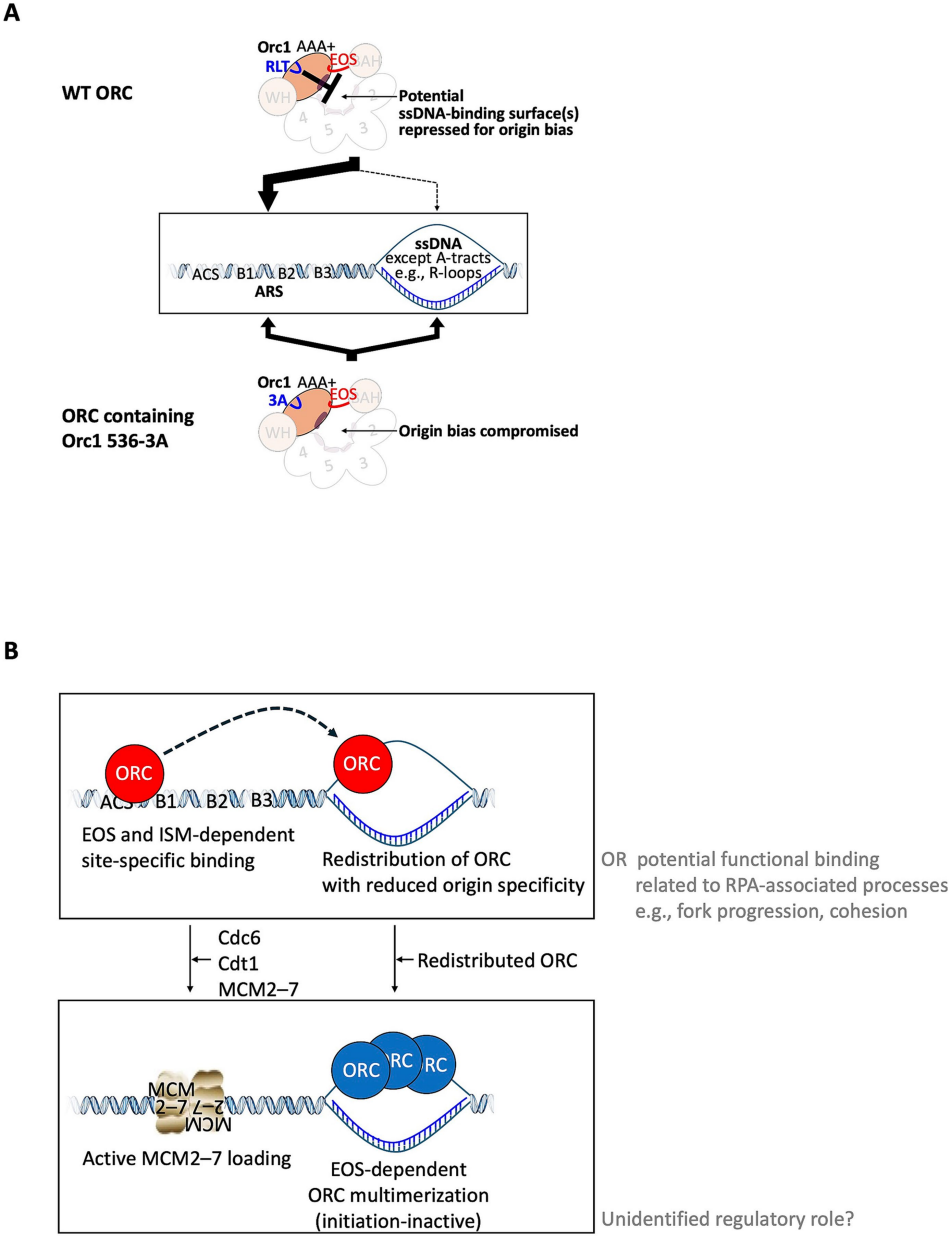
Model for Orc1 R536–T538–dependent regulation of ORC origin bias and ssDNA binding. **(A)** Model for Orc1 residues R536–T538-dependent repression of ORC–ssDNA binding. In wild-type cells, Orc1 residues R536–T538 (RLT) repress the ssDNA-binding activity of ORC, thereby biasing ORC toward stable, origin-bound dsDNA states through EOS-dependent recognition of the ACS. In ORC containing Orc1 536-3A, this repression is compromised by alanine substitutions (3A), allowing the ssDNA-binding activity to become unmasked (except for poly dA tracts). ORC binding to ssDNA *in vitro* is mutually exclusive with origin dsDNA binding (Lee et al., 2000). **(B)** Model for redistribution of ORC to ssDNA-forming loci. Upon loss of stable, site-specific origin binding, ORC is redistributed to ssDNA-forming loci, including transcription-associated regions such as R-loops, where exposed ssDNA permits ORC association. ORC molecules accumulated at these loci can multimerize in an EOS-dependent manner, forming ORC–ssDNA complexes that are unlikely to support replication initiation. A subset of ORC–ssDNA interactions may represent potentially functional binding events related to RPA-associated processes, such as replication fork progression or sister chromatid cohesion, with a possible as-yet-unidentified regulatory role (gray).

We also found that ORC and RPA have overall comparable binding affinities to ssDNA *in vitro*. Within this overall range, ORC–ssDNA binding shows a clear base-composition preference, particularly against poly dA ssDNA (Figure 2). Considering that ORC and RPA partially co-enrich at ssDNA-forming genomic regions (Figures 2E–G; Supplementary Figures 3, 9), our data support a model in which ORC can bind both dsDNA and ssDNA, but residues R536–T538-dependent binding to origin dsDNA biases ORC toward origin-bound states rather than stable ssDNA binding, thereby stabilizing origin-specific ORC–dsDNA binding so that MCM2–7 can be loaded (Figure 10A). Indeed, ORC binding to ssDNA *in vitro* is mutually exclusive with origin dsDNA binding (Lee et al., 2000). Consistent with impaired specificity of origin binding, ORC containing Orc1 536-3A also significantly reduces ARS DNA-dependent repression of ORC ATPase activity (Figure 4). Reduced affinity for poly dA ssDNA, similar to that shown in Figure 2, has been reported for the human homologous recombination protein RAD51 (Paoletti et al., 2020), suggesting that properties of ssDNA (e.g., flexibility as shown for RAD51) are critical determinants influencing ORC–ssDNA interactions, while not excluding additional sequence-dependent contributions.

Although dA/dT-rich ACSs and B elements are prominent features of budding yeast replication origins, their role should be distinguished from direct sequence recognition of ssDNA by ScORC. *In vivo*, ARSs exist predominantly as dsDNA, and ORC binds these elements as a duplex. The short A/T-rich elements within ARSs are unlikely to function as direct ssDNA substrates given that ORC–ssDNA binding *in vitro* requires substrates of several tens of nucleotides (Lee et al., 2000). Instead, poly dA:dT tracts are known to modulate local chromatin architecture by disfavoring nucleosome occupancy and increasing accessibility of adjacent origin elements (Eaton et al., 2010). Moreover, different ssDNA-binding proteins exhibit distinct preferences for poly dA and poly dT tracts, with some proteins favoring poly dT over poly dA, whereas others can utilize poly dA-containing ssDNA (e.g., Kim et al., 1992; Nakagawa and Kolodner, 2002; Tubbs et al., 2018; Paoletti et al., 2020). Our biochemical data indicate that ORC shows reduced binding to poly dA relative to poly dT ssDNA (Figure 2). Genome-wide analyses further show that highly transcribed genes preferentially form dA-rich:rU-rich DNA–RNA hybrids with displaced dT-rich single strands (Wahba et al., 2016). This bias raises the possibility that ORC may bind transcription-associated displaced dT-rich ssDNA while avoiding dA-rich template strands. Thus, the presence of poly dA:dT tracts at ARSs does not contradict our observation that ORC disfavors poly dA ssDNA. In contrast, ORC is thought to dissociate from the ARS upon loading of the MCM2–7 helicase core, a precursor of the active CMG helicase, onto dsARS. Therefore, ORC is not currently considered to function during duplex unwinding or elongation at replication forks (Bell and Labib, 2016). This reinforces the notion that ORC–ssDNA binding represents a mechanistically distinct mode from canonical origin licensing and replication elongation.

Our results show that ORC containing Orc1 536-3A has greater affinity for ssDNA than wild-type ORC (Figure 6). This result was initially unexpected given that a mutation in the DnaA H-motif abolishes ssDNA-binding activity (Ozaki et al., 2008). A plausible explanation for this is a structural difference: the ISM in DnaA is an antiparallel *α*-helix with a short connecting loop, whereas the corresponding region of *D. melanogaster* Orc1 is a parallel helix with a longer loop (Bleichert et al., 2015). Thus, the ISM-related region in ORC appears to have differentiated from bacterial DnaA, thereby providing a mechanistic basis for the negative regulation of ORC–ssDNA binding. Consistent with this, ORC containing Orc1 536-3A forms aberrant dsDNA complexes with altered electrophoretic mobility, suggesting an altered DNA-binding mode (Figure 5).

It has been a longstanding open question why ORC has ssDNA-binding activity even though ScMCM2–7 is loaded onto dsDNA. Although ORC can bind ssDNA *in vitro* (Lee et al., 2000; Kawakami et al., 2015), our data strongly suggest that ORC–ssDNA binding is likely undesirable *in vivo* under these conditions, because inappropriate ORC binding to ssDNA could be detrimental for specific origin selection. This interpretation is reasonable because ssDNA could be transiently exposed at certain chromosomal loci even in the absence of DNA replication, including transcription-associated processes such as R-loop formation. Mitotic condensin is enriched at highly transcribed loci and targets transcription-associated unwound DNA structures (Sutani et al., 2015). Condensin preferentially binds ssDNA and counteracts RPA *in vitro* (Akai et al., 2011). Along with genomic colocalization between condensin and ORC at such regions (Kawakami et al., 2019), these observations are consistent with the idea that transcription-coupled ssDNA exposure may influence the chromatin association of multiple ssDNA-binding factors, including ORC, during M and G1 phases (Hsiung et al., 2016). Bacterial condensin also prefers ssDNA to dsDNA and selects ribosomal genes as its loading sites (Niki and Yano, 2016; Yano et al., 2022), suggesting that ssDNA-mediated targeting of condensin to highly transcribed genes may be conserved across domains of life.

Consistent with this idea, previous ChIP-chip studies identified numerous ORC-binding sites within highly transcribed ORFs lacking ACSs, and only a subset of these sites overlapped with MCM binding (Shor et al., 2009), indicating that ORC binding outside canonical origins is often uncoupled from efficient MCM2–7 loading. Therefore, the central question may be reframed from why ORC binds ssDNA to why ORC has evolved mechanisms to avoid functional ssDNA binding, thereby minimizing inappropriate MCM2–7 loading. Based on our biochemical, genetic, and chromatin-based analyses, we propose a model in which Orc1 residues R536–T538 function as a negative regulatory element that inhibits one or more ssDNA-binding surfaces within ORC, thereby limiting stable ORC binding to ssDNA-exposing regions outside replication origins (Figure 10). Any such non-origin ssDNA binding *in vivo* may be biased away from poly dA tracts, given that ORC–ssDNA binding is disfavored for poly dA ssDNA *in vitro* (Figure 2). In parallel, R536–T538 may contribute to origin specificity through indirect structural mechanisms. One possibility is that R536–T538 regulate the conformation or dynamics of a neighboring IDR within Orc1, thereby modulating the binding between the Orc1 EOS region and the ACS. Alternatively, or in addition, R536–T538 may influence non-sequence-specific contacts between Orc1 and the DNA backbone, such as those mediated by the adjacent residue W539, which contacts the DNA phosphate in ORC–ARS structures. Such effects may include fine-tuning or stabilization of site-specific ORC association with ds ARS DNA (Figure 1A), even if the present data do not distinguish direct dsDNA contacts from indirect or allosteric effects. Subtle modulation of these backbone interactions could indirectly affect the stability of EOS- and Orc4-mediated ACS recognition, without altering sequence-specific contacts *per se*. Through these combined mechanisms, R536–T538 may act as a coupling element that represses ssDNA binding while promoting origin-specific dsDNA binding, thereby contributing to faithful origin selection *in vivo*. The enhanced ssDNA binding and higher-order ORC–ssDNA complex formation observed for ORC containing Orc1 536-3A (Figure 6) raise the possibility that excessive ssDNA binding may shift ORC toward a functionally inactive state, although the impact of such multimerization on initiator activity remains to be determined.

Given that genomic DNA in all organisms can locally form ssDNA independently of DNA replication, the untimely binding of the replication initiator to ssDNA is likely a conserved challenge across species. Because the ISM-related region in Orc1 is highly conserved among eukaryotic homologs, including the human protein (Figure 3; Supplementary Figure 4), we suggest that the negative regulatory mechanism of ORC–ssDNA binding mediated by the Orc1 ISM-related region may be shared among eukaryotes. This idea is consistent with the fact that replication origins in higher eukaryotes are enriched at transcriptional promoters (Cadoret et al., 2008; Sequeira-Mendes et al., 2009), where transcription-associated DNA unwinding and ssDNA exposure are frequent. Moreover, replication origins in human and mouse cells are associated with G4 consensus motifs (Besnard et al., 2012; Cayrou et al., 2015), for which the complementary strands are invariably C-rich. Consistent with this, human Orc1 has moderate affinity for C-rich ssDNA *in vitro*, as well as higher affinity for G4-forming ssDNA (Hoshina et al., 2013), supporting the idea that ORC–ssDNA binding is a conserved mechanism that requires tight regulation to prevent non-origin association in eukaryotic genomes.

Conceptually similar strategies may extend to other DNA-binding proteins. The tumor suppressor p53 binds both ssDNA ends and dsDNA (Selivanova et al., 1996) and regulates its DNA-binding specificity through intramolecular autoinhibition involving distinct domains, as well as through post-translational modifications (Gu and Roeder, 1997; Cain et al., 2000; He et al., 2019). Likewise, iteron-based plasmid replication systems feature initiator titration or “handcuffing” mechanisms to limit inappropriate initiator activity (Chattoraj, 2000). These examples illustrate that repressing inappropriate DNA-binding modes either by active exclusion or by diversion into inactive DNA-bound states can be an effective strategy to achieve specific and functional DNA binding. By analogy, the ORC-dependent regulation described here may represent a broadly applicable principle by which genome-associated proteins achieve functional specificity despite possessing multiple DNA-binding modes. In certain cases, near-palindromic and G-rich sequences (Supplementary Figure 10) could contribute to regulatory processes by transiently adopting non-B DNA conformations or by facilitating local ssDNA exposure (Figure 10B, gray). We do not exclude the possibility that ORC may have an as-yet-unidentified role in replication-coupled processes, potentially including functions that are coordinated with, but distinct from, RPA-dependent mechanisms associated with replication fork progression or the establishment of sister chromatid cohesion (Figure 10B, gray). Whether ORC–ssDNA interactions contribute to such processes remains to be elucidated.

## Data Availability

The original contributions presented in the study are included in the article/Supplementary material, further inquiries can be directed to the corresponding author.
