# Supplementary material for: An Orc1 initiator-specific motif (ISM)-related region limits ORC–ssDNA binding and promotes replication origin specificity in budding yeast
Source: Front Microbiol. 2026 Apr 22;17:1778270. doi: 10.3389/fmicb.2026.1778270 (PMC13144144; doi:10.3389/fmicb.2026.1778270)
Supplement: Supplementary file 1 [file Data_sheet_1.pdf]

Supplementary Material

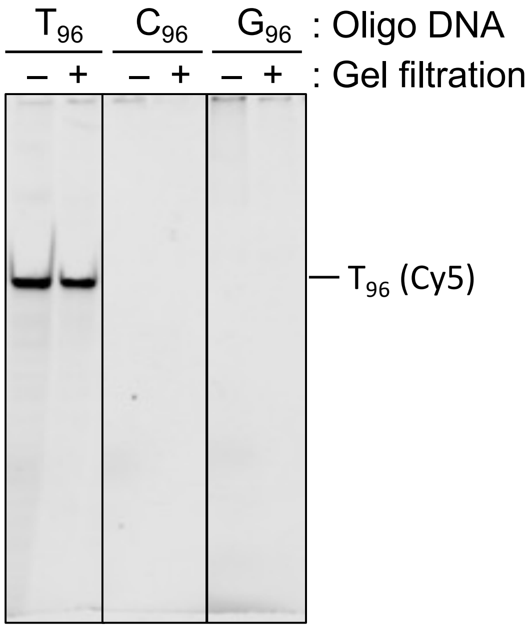

Supplementary Figure 1. **Cy5 labeling of oligonucleotides using terminal transferase.** Oligonucleotides (10 pmol) were incubated in a 20 µl reaction containing 0.5 mM Cy5-ddUTP and 400 units of terminal transferase. Reactions were stopped by the addition of 0.8 µl of 0.5 M EDTA and subjected to gel filtration using a Micro Bio-Spin 6 column (Bio-Rad). The eluate fraction excluding free Cy5-ddUTP was collected. Cy5 signals in samples before (–) and after (+) gel filtration were analyzed by 10% PAGE and detected with a LAS-4010 imager. All lanes were derived from the same gel.

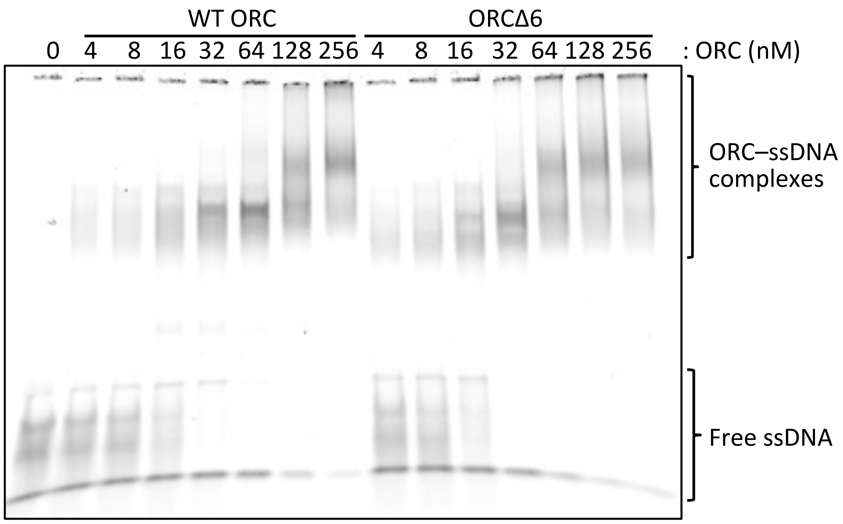

Supplementary Figure 2. **Orc6 has no major effect on ORC–ssDNA binding.**
EMSA was performed with the indicated amounts of wild-type ORC and ORC lacking Orc6 (ORCΔ6) using Cy5-labeled DL15 ssDNA in the presence of 1 mM ATP. Reaction mixtures were resolved by 4% native PAGE and analyzed using a Typhoon Trio+ imager.

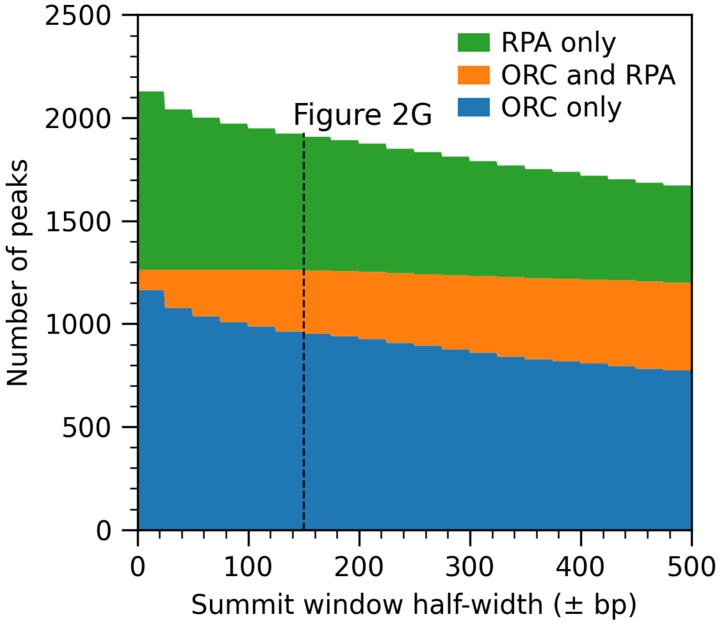

Supplementary Figure 3. Effect of summit-centered window size on ORC–RPA overlap. Quantitative overlap analyses were performed using SICER2 peak sets passing a fixed BH-adjusted FDR cutoff (≤5%). Overlap was evaluated by varying the summit-centered window size from 0 to 500 bp. The window size used for Figure 2G and the corresponding overlap value are indicated by a dotted line for reference.

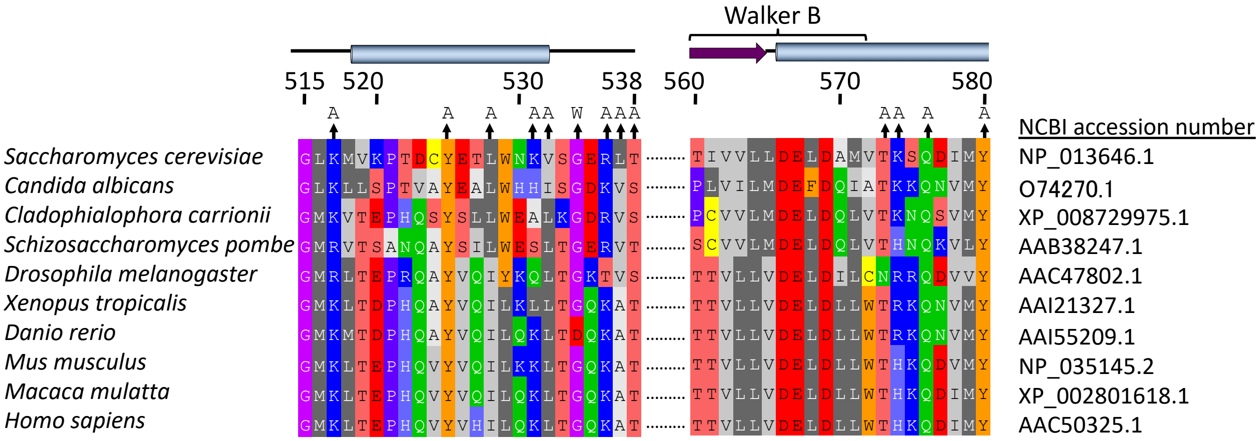

Color	Residue	Description
	C	Cysteine
	DE	Acidic
	KR	Basic
	H	Slightly Basic
	FWY	Aromatic
	G	Glycine
	P	Proline
	NQ	Amido-sidechains
	A	Alanine
	IV	Hydrophobic 1
	LM	Hydrophobic 2
	ST	Hydroxy containing
	–	Gap

Supplementary Figure 4. Multiple sequence alignment of Orc1 homologs from representative model species and pathogens.

A representative portion of the alignment shown in Figure 3B is presented, with residues colored according to the legend. NCBI accession numbers are indicated.

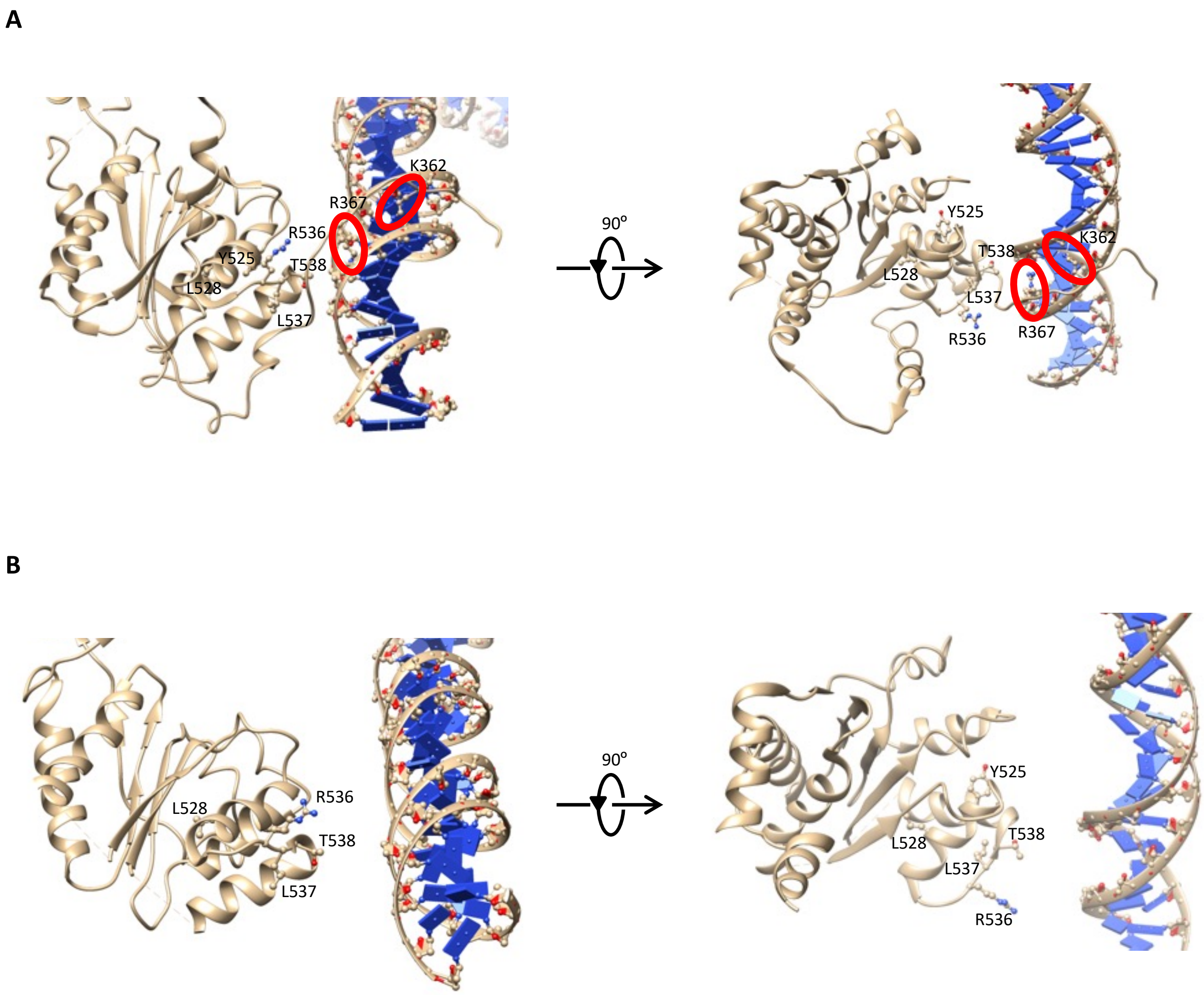

Supplementary Figure 5. ScOrc1 residues R536–T538 are positioned distal to dsARS DNA in ORC–ARS and OCCM complexes.

Portions of the resolved structures of Orc1 and ARS DNA in the ScORC–ARS complex (PDB: 5ZR1) (**A**) and the ScORC–Cdc6–Cdt1–MCM2–7 complex (PDB: 5UDB) (**B**) are shown (Yuan et al., 2017; Li et al., 2018). Images on the left are displayed in a similar orientation and style as in Figure 3C. Orc1 residues R536–T538 and ARS DNA, as well as Y525, L528, and EOS residues K362 and R367 (where structurally resolved), are shown as ball-and-stick models.

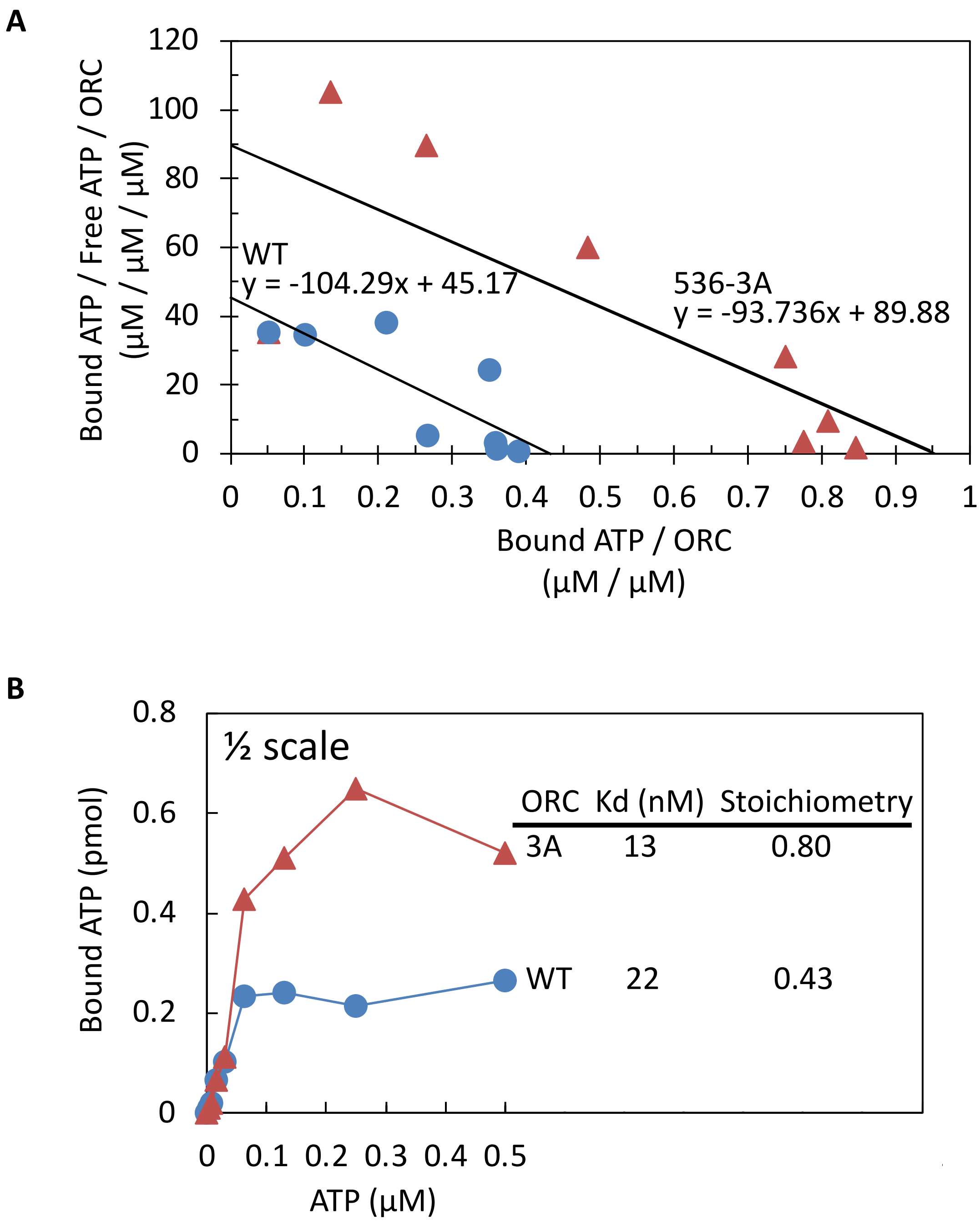

Supplementary Figure 6. **ORC–ATP binding analysis.**

(**A**) The Kd and stoichiometry of ORC–ATP binding (Figure 4B) were determined by Scatchard analysis. The slope and *x*-intercept of the linear regression correspond to −1/Kd and the stoichiometry, respectively. (**B**) ATP-binding assays were performed at half scale relative to those shown in Figure 4B. The Kd and stoichiometry were similarly deduced.

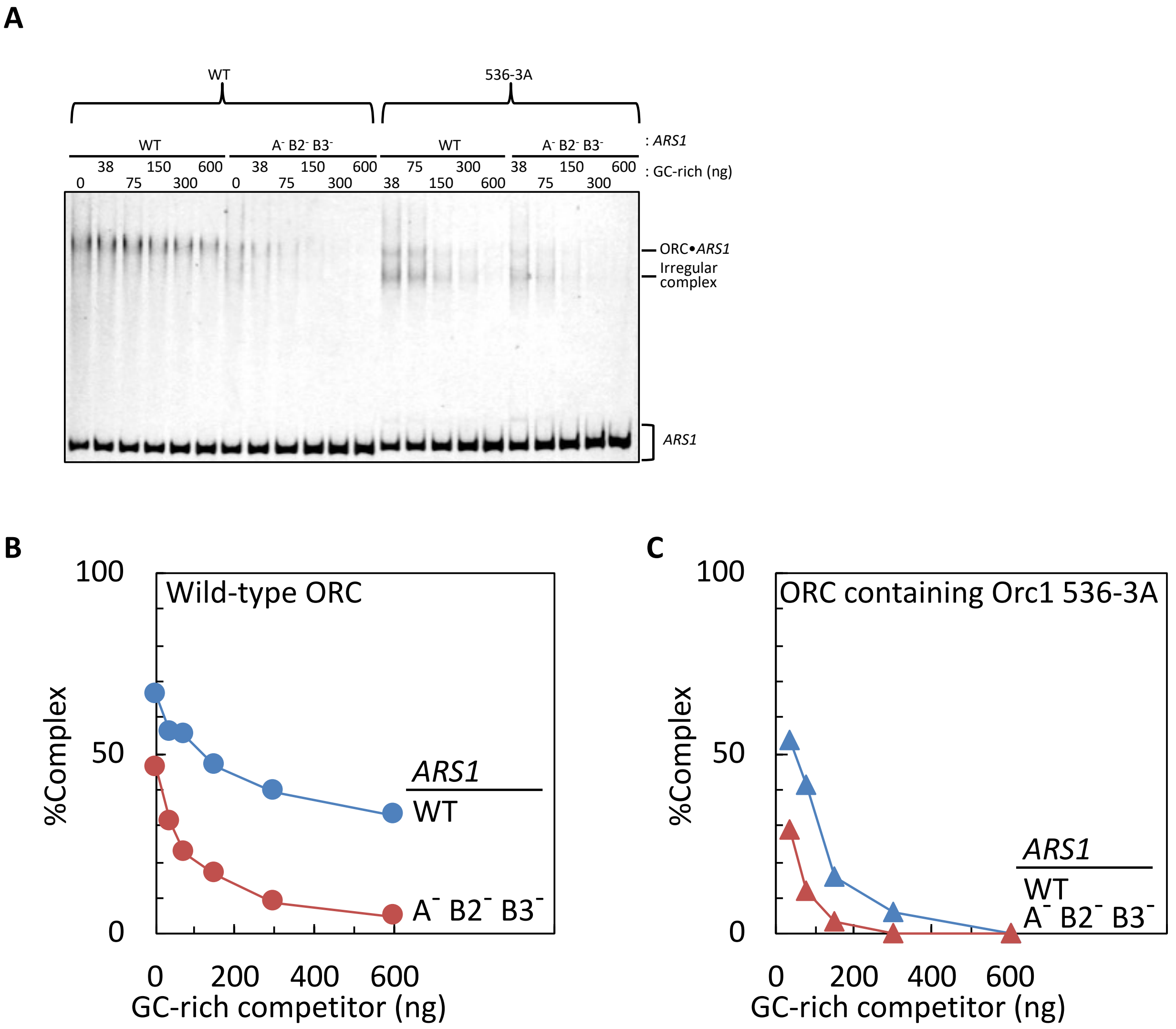

Supplementary Figure 7. **DNA-binding specificity of ORC containing Orc1 536-3A.** (**A**) EMSA was performed with fixed ORC concentration (2 nM) and increasing GC-rich competitor DNA. (**B and C**) Quantification of panel A for wild-type ORC (**B**) or ORC containing Orc1 536-3A (**C**).

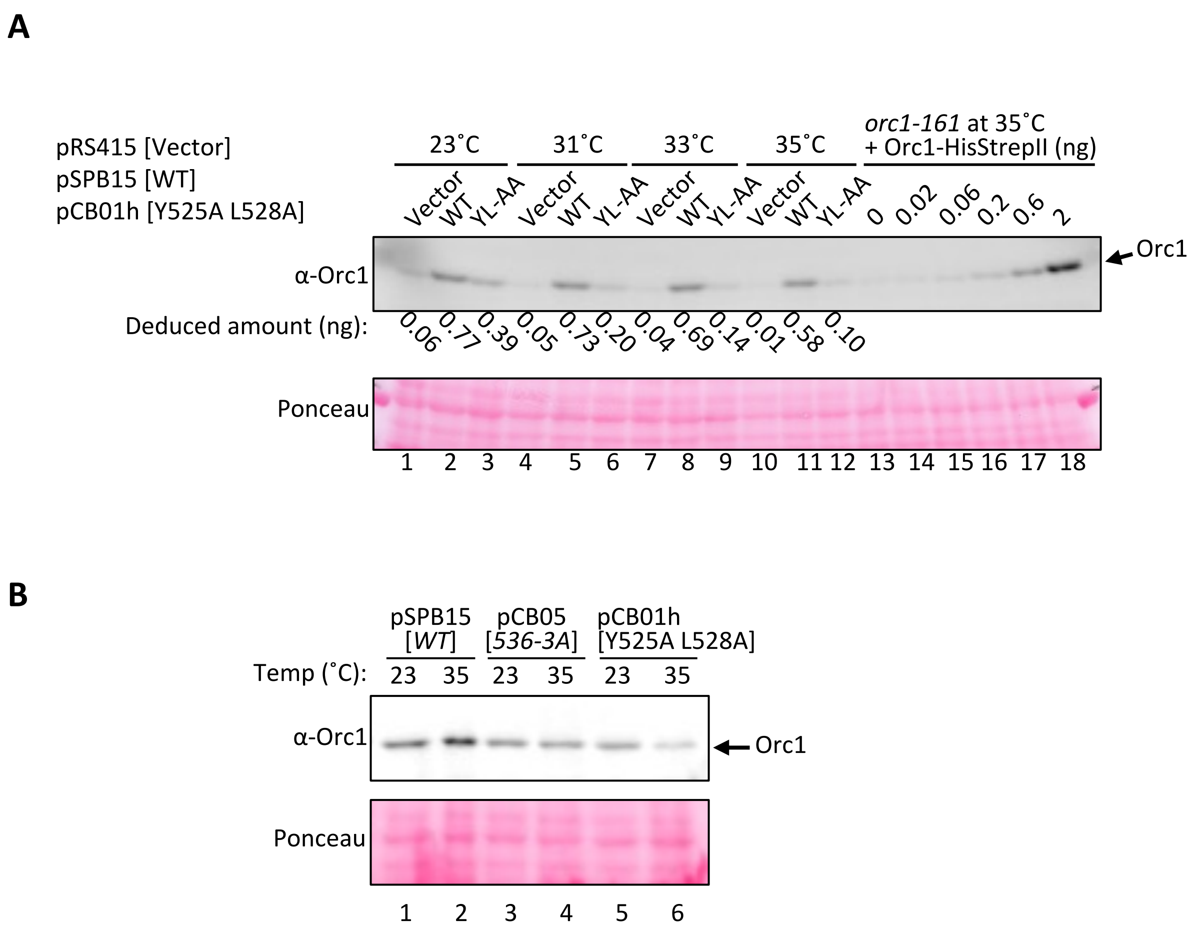

Supplementary Figure 8. Cellular levels of mutant Orc1 proteins.
OAy66 (*orc1-161*) cells harboring the indicated plasmids were grown at 23˚C and subsequently incubated at the indicated temperatures for 2 h. Whole cells were lysed and analyzed by 5% SDS-PAGE, followed by Western blotting using an anti-Orc1 antibody. Ponceau staining was performed as a loading control. In panel **A**, the estimated cellular amounts of Orc1 are indicated.

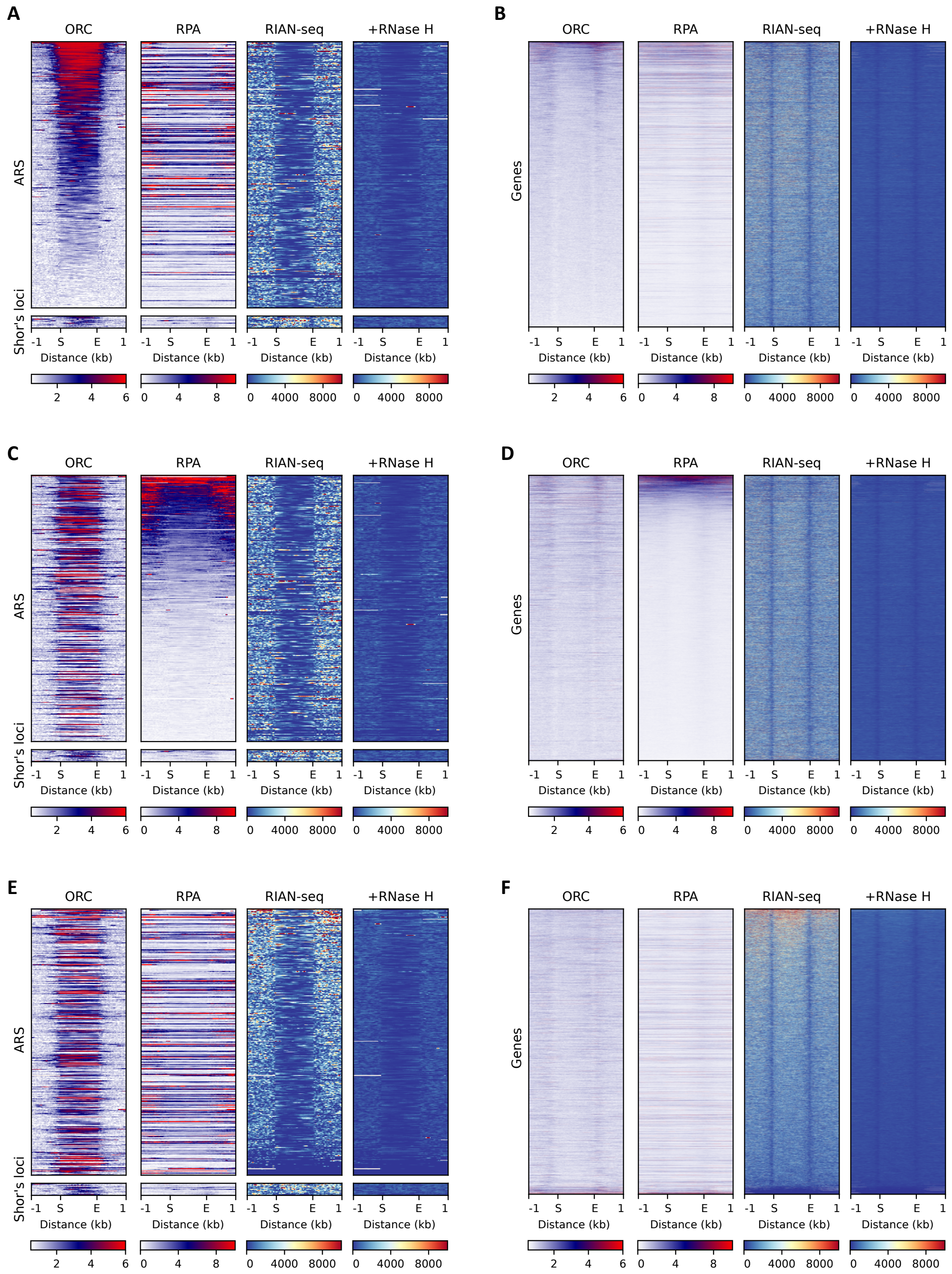

Supplementary Figure 9. Genomic co-localization of ORC and RPA in annotated regions. Heatmaps of ORC, RPA, RIAN-seq, and RNase H–treated RIAN-seq signals are shown for annotated ARSs (n = 543) together with genes previously reported as ACS-independent ORC-binding loci by Shor et al. (n = 16; see text for details) in panels **A**, **C**, and **E**, and for all annotated genes (n = 6,585) in panels **B**, **D**, and **F**. In panels **A**, **C**, and **E**, ARSs are shown in the upper portion and Shor's loci in the lower portion. For visualization, annotated regions were scaled to a uniform length and plotted from the start (S) to the end (E). Regions were sorted in descending order based on the mean ORC signal (**A** and **B)**, the mean RPA signal (**C** and **D)**, and the mean RIAN-seq signal (**E** and **F).**

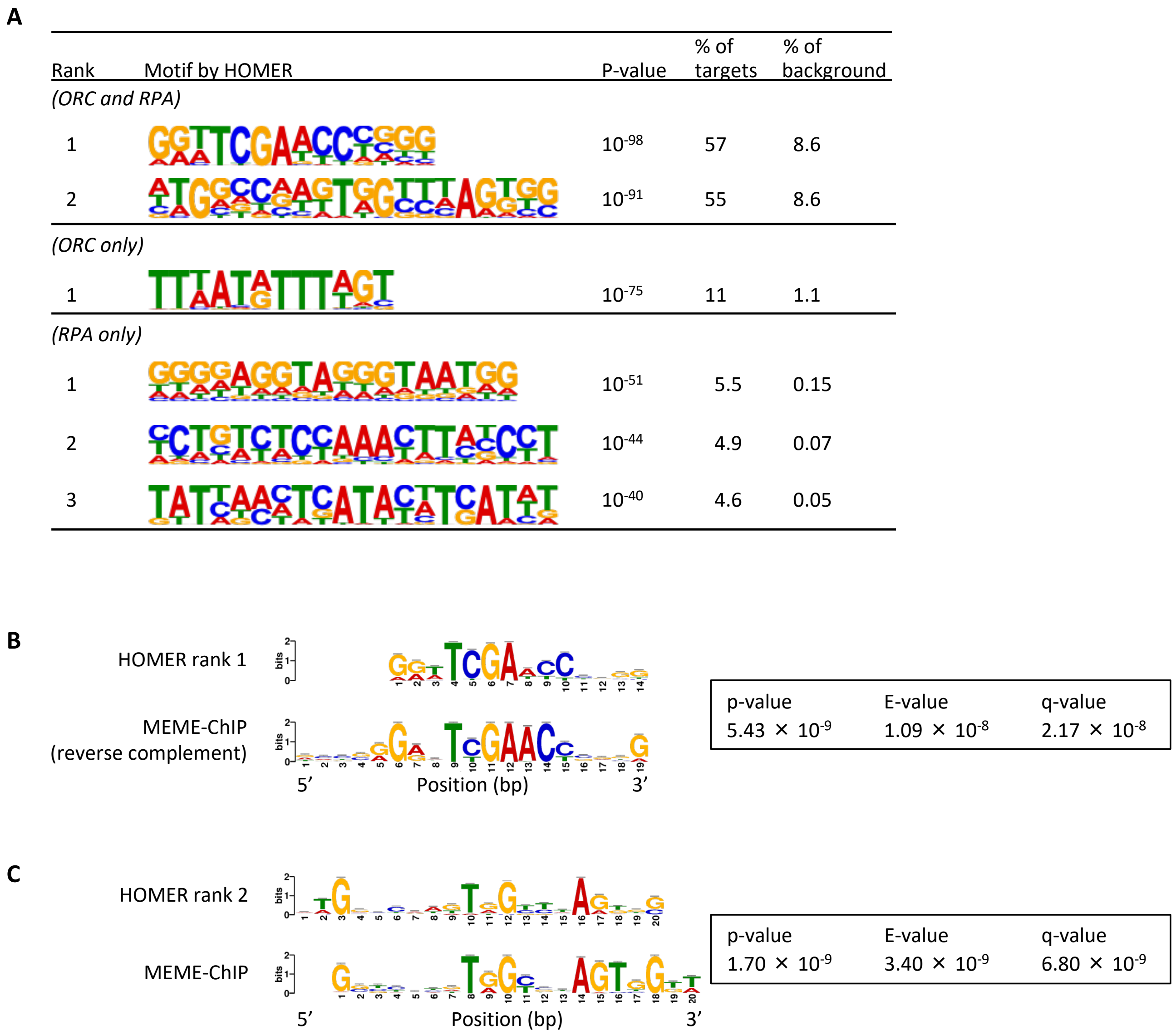

Supplementary Figure 10. Sequence analyses of genome-wide ORC–RPA co-localized loci. (**A**) Sequence logos of *de novo* motifs identified in ORC–RPA co-localized ChIP-seq peak sets using HOMER. Motifs significantly enriched relative to ORC-only and RPA-only peak sets are shown. For comparison, the top motif hit obtained from the ORC-only peak set and the top three motifs from the RPA-only peak set are also shown. (**B, C**) Sequence logos and Tomtom similarity analyses of rank 1 (**B**) and rank 2 (**C**) motifs identified in ORC–RPA co-localized loci. Motifs independently identified by MEME-ChIP were compared with the corresponding HOMER-derived motifs using Tomtom. Reported p-, E-, and q-values indicate the statistical significance of PWM similarity.

Supplementary Table 1. Oligonucleotides used in this study.

Name	Sequence (5'→3')
DL15	ACCTGTCGTGCCAGCTGCATTAATGAATCGCGACCCCCCATTCAAG AACAGCAAGCAGCATTGAGAACTTTGGAATCCAGTCCCTCTTCCAC CTGC
HK461	T_{79}
RM05	A_{96}
RM06	T_{96}
RM07	C_{96}
RM08	G_{96}
HK467	$(CA)_{48}$
HK468	$(GA)_{48}$

Supplementary Table 2. Yeast strains used in this study.

Name	Relevant genotype[a]	Reference or source
YB838	*orc1::hisG* pSPB16 (*ORC1 ARS CEN URA3*)	(Kawakami et al., 2015)
OAy66	*orc1Δ::orc1-161(URA3)*	Stephen P. Bell
OAy422	OAy66 *bar1::LEU2*	(Kawakami et al., 2015)
YHK26	OAy422 *his3::pRS403*	(Kawakami et al., 2015)
YHK33	OAy422 *his3::ORC1-*His_{12} *(HIS3)*	(Kawakami et al., 2015)
YHK35	OAy422 *his3::orc1 R367A-*His_{12} *(HIS3)*	(Kawakami et al., 2015)
YCB3A	OAy422 *his3::orc1 536-3A-*His_{12} *(HIS3)*	This study
YCB415	OAy66 pRS415	This study
YCBB15	OAy66 pSPB15 (*ORC1 ARS CEN LEU2*)	This study
YCB01-h	OAy66 pCB01-h (pSPB15 *Y525A L528A*)	This study
YCB05	OAy66 pCB05 (pSPB15 *536-3A*)	This study
YCB20	OAy422 pRS413	This study
YCB21	OAy422 pSPB13 (*ORC1 ARS CEN HIS3*)	This study
YCB22	OAy422 pCB05-13 (pSPB13 *536-3A*)	This study

[a]All yeast strains are derivatives of W303-1A (*MAT***a** *leu2-3,112 his3-11,15 trp1-1 can1-100 ade2-1 ura3-1*).

Supplementary Table 3. Purification table of ORC containing Orc1 536-3A.

Fraction	Step	Volume	Concentration	Protein	Yield
		ml	*mg/ml*	*mg*	*%*
I.	Lysate	9.0	15	135	[100]
II.	HisTrap	10	0.49	4.9	3.7
III.	SP Sepharose (first[a])	1.0	1.3	1.3	1.4
IV.	Superdex 200	6.0	0.098	0.59	0.44
V.	SP Sepharose (second[b])	0.25	0.70	0.18	0.13

[a] 0.5 ml bed volume

[b] 0.25 ml bed volume

Supplementary Table 4. Conserved k-mer enrichment patterns at ORC–RPA rank 1 and rank 2 loci.[a]

k-mer	Mismatches allowed	Target region (hit / total)	Control (hit / total)	Odds ratio	Fisher's exact test p	Dinucleotide shuffle p_{emp} [b]
(ORC–RPA rank 1 loci vs. ORC-only and RPA-only control regions)						
GGTTCGAACC	1	59 / 293 (20%)	24 / 1604 (1.5%)	17	3.2×10^{-16}	$< 1 \times 10^{-4}$
	2	156 / 293 (53.2%)	146 / 1604 (9.10%)	11.3	1.54×10^{-15}	$< 1 \times 10^{-4}$
(ORC–RPA rank 2 loci vs. ORC-only and RPA-only control regions)						
GN_6TGGCN_2AGTGG	0	11 / 293 (3.8%)	3 / 1604 (0.2%)	2×10^1	2×10^{-7}	$< 1 \times 10^{-4}$
	1	56 / 293 (19%)	29 / 1604 (1.8%)	13	1.0×10^{-15}	$< 1 \times 10^{-4}$
	2	91 / 293 (31%)	131 / 1604 (8.2%)	5.1	1.5×10^{-15}	$< 1 \times 10^{-4}$
$CCACTN_2GCCAN_6C$ (reverse complement)	0	15 / 293 (5.1%)	3 / 1604 (0.2%)	3×10^1	3×10^{-10}	$< 1 \times 10^{-4}$
	1	51 / 293 (17%)	29 / 1604 (1.8%)	11	3.7×10^{-16}	$< 1 \times 10^{-4}$
	2	97 / 293 (33%)	125 / 1604 (7.8%)	5.9	1.5×10^{-15}	$< 1 \times 10^{-4}$

[a] Enrichment of the near-palindromic GC-rich core k-mer (rank 1) and the G-rich motif (rank 2) was evaluated using Fisher's exact test and dinucleotide-preserving permutation tests. For each motif, patterns capturing the 10 fixed positions within the 10-bp core (rank 1) or the 18-bp module (rank 2; 10/18 positions fixed) were used. Mismatch allowance (0–2) refers to mismatches counted only at fixed (non-degenerate) positions. For rank 1, no exact matches to the 10-bp core sequence were detected in the target set and were therefore omitted.

[b] Empirical p-values were calculated as $(r + 1)/(n + 1)$, where n = 10,000 permutations.

Supplementary Table 5. Motif scanning analysis of rank 1 and rank 2 motifs at Shor's reported ORC-binding loci.[a]

Chromosome	Start	Stop	Strand	Score	p-value	q-value	Matched_sequence	Annotation
(Rank 1)								
chrXII	838341	838355	+	10.4737	7.49×10^{-6}	0.301	A**GGTT**T**GAAC**TTGAG	*ILV5*
chrVIII	451765	451779	-	2.94737	6.37×10^{-5}	0.77	A**G**AA**T**G**GAAC**TGGCA	*ENO2*
chrVII	1001365	1001379	-	2.94737	6.37×10^{-5}	0.77	A**G**AA**T**G**GAAC**TGGCA	*ENO1*
chrII	477901	477915	-	1.47368	9.57×10^{-5}	0.77	A**G**T**TTCGAAC**TTCCA	*TEF2*
chrXVI	700824	700838	-	1.47368	9.57×10^{-5}	0.77	A**G**T**TTCGAAC**TTCCA	*TEF1*
(Rank 2)								
chrXI	326728	326747	-	9.88158	3.05×10^{-5}	0.553	T**G**GCACAG**TGG**TCAGAGT**G**T	*FBA1*
chrII	478857	478876	+	7.80263	6.37×10^{-5}	0.553	C**G**CTGCTT**TGG**TCA**AGT**TCG	*TEF2*
chrXVI	701780	701799	+	7.80263	6.37×10^{-5}	0.553	C**G**CTGCTT**TGG**TCA**AGT**TCG	*TEF1*
chrXV	445067	445086	-	7.57895	6.86×10^{-5}	0.553	A**G**AGTATT**TGG**TGA**A**AGCCT	*RPL3*
chrX	338392	338411	+	7.38158	7.32×10^{-5}	0.553	T**G**CTTACA**TGG**TCA**AGT**ACG	*TDH1*

a FIMO was run using MEME-derived PWMs of rank 1 and rank 2 motifs with default significance threshold settings (p-value ≤ 1×10^{-4}). All detected motif matches are listed, with gene names manually annotated. Sequences matching the core sequences of rank 1 (GGTTCGAACC) and rank 2 motifs (GN_6TGGCN_2AGTGG), as defined in Supplementary Table 4, are shown in bold red. Note that none of the detected matches satisfied a BH–adjusted FDR (q-value) ≤ 0.05.

Supplementary references

Kawakami, H., Ohashi, E., Kanamoto, S., Tsurimoto, T., and Katayama, T. (2015). Specific binding of eukaryotic ORC to DNA replication origins depends on highly conserved basic residues. *Sci. Rep.* 5, 14929. doi: 10.1038/srep14929

Li, N., Lam, W. H., Zhai, Y., Cheng, J., Cheng, E., Zhao, Y., et al. (2018). Structure of the origin recognition complex bound to DNA replication origin. *Nature* 559, 217–222. doi: 10.1038/s41586-018-0293-x

Yuan, Z., Riera, A., Bai, L., Sun, J., Nandi, S., Spanos, C., et al. (2017). Structural basis of Mcm2–7 replicative helicase loading by ORC–Cdc6 and Cdt1. *Nat. Struct. Mol. Biol.* 24, 316–324. doi: 10.1038/nsmb.3372
